# Supplementary material for: Brief disruption of activity in a subset of dopaminergic neurons during consolidation impairs long-term memory by fragmenting sleep
Source: eLife. 2026 Sep 23;14:RP104862. doi: 10.7554/eLife.104862 (PMC13600697; doi:10.7554/eLife.104862)
Supplement: Supplementary file 1 [file elife-104862-supp1.pdf]

Table 1. One way ANOVA for memory and neural activity tests (Related to Figs. 3, 6, 7, 9-11, S1, S3)

	ANOVA	DFn, DFd	F	p	post-hoc test	Comparisions	n1	n2	Mean Diff.	Adjusted p Value	Summary
Fig. 3A	Kruskal-Wallis	3, 27	16.75	0.0002	Dunn's	R58E02/shi^{ts1} vs R58E02/+	9	8	-51.69	0.0033	**
						R58E02/shi^{ts1} vs shi^{ts1}/+	9	10	-56.39	0.0003	***
Fig. 3B	One way ANOVA	2, 40	12.04	＜0.0001	Sidak's	c316/shi^{ts1} vs c316/+	14	14	-25.95	0.0003	***
						c316/shi^{ts1} vs shi^{ts1}/+	14	15	-26.15	0.0002	***
Fig. 3C	One way ANOVA	2, 90	7.534	0.0009	Sidak's	VT064246/shi^{ts1} vs VT064246/+	28	35	-10.81	0.0158	*
						VT064246/shi^{ts1} vs shi^{ts1}/+	28	30	-15.67	0.0005	***
Fig. 3D	One way ANOVA	2, 20	0.384	0.6861	Sidak's	c316/shi^{ts1} vs c316/+	9	6	-6.252	0.7470	ns
						c316/shi^{ts1} vs shi^{ts1}/+	9	8	0.8992	0.9928	ns
Fig. 3E	One way ANOVA	2, 47	12.90	＜0.0001	Sidak's	R58E02/dTrpA1 vs R58E02/+	16	18	-31.73	＜0.0001	****
						R58E02/dTrpA1 vs dTrpA1/+	16	16	-23.71	0.0016	**
Fig. 3F	One way ANOVA	2, 44	3.187	0.0510	Sidak's	R58E02/dTrpA1 vs R58E02/+	15	18	-13.21	0.0331	*
						R58E02/dTrpA1 vs dTrpA1/+	15	14	-9.268	0.2037	ns
Fig. 3G	One way ANOVA	2, 44	3.827	0.0293	Sidak's	R58E02/shi^{ts1} vs R58E02/+	18	15	-4.409	0.6971	ns
						R58E02/shi^{ts1} vs shi^{ts1}/+	18	14	-16.03	0.0185	ns
Fig. 6B	One way ANOVA	2, 46	4.201	0.0211	Sidak's	R15A04/dTrpA1 vs R15A04/+	16	17	-18.97	0.0386	*
						R15A04/dTrpA1 vs dTrpA1/+	16	16	-19	0.0417	*
Fig. 6D	Kruskal-Wallis	3, 54	2.163	0.3391	Dunn's	R48B04/dTrpA1 vs R48B04/+	21	17	-0.444	>0.9999	ns
						R48B04/dTrpA1 vs dTrpA1/+	21	16	-7.082	0.3498	ns
Fig. 7A	One way ANOVA	2, 44	5.597	0.0068	Sidak's	MB299B/dTrpA1 vs MB299B/+	14	16	-25.14	0.0073	**
						MB299B/dTrpA1 vs dTrpA1/+	14	17	-22.45	0.0159	*
Fig. 7B	One way ANOVA	3, 54	11.72	0.0029	Sidak's	MB043B/dTrpA1 vs MB043B/+	18	20	-13.69	0.0148	*
						MB043B/dTrpA1 vs dTrpA1/+	18	16	-17.2	0.0029	**
Fig. 7C	One way ANOVA	2, 36	9.9375	0.0005	Sidak's	MB299Bshi^{ts1} vs MB299B/+	14	11	-24.98	0.0034	**
						MB299B/shi^{ts1} vs shi^{ts1}/+	14	14	-27.41	0.0007	***
Fig. 7D	Kruskal-Wallis	3, 65	1.966	0.3742	Dunn's	MB043B/shi^{ts1} vs MB043B/+	24	16	-5.969	0.6560	ns

						MB043B/shi^{ts1} vs shi^{ts1}/+	24	25	-7.23	0.3617	ns
Fig. 7E	One way ANOVA	2, 37	4.849	0.0135	Sidak's	MB299B/dTrpA1 vs MB299B/+	12	11	-18.26	0.0378	*
						MB299B/dTrpA1 vs dTrpA1/+	12	17	-19.7	0.0116	*
Fig. 7F	One way ANOVA	2, 56	0.5049	0.6063	Sidak's	MB299B/dTrpA1 vs MB299B/+	18	18	-2.053	0.8939	ns
						MB299B/dTrpA1 vs dTrpA1/+	18	23	-4.574	0.5417	ns
Fig. 7G	Kruskal-Wallis	3, 28	2.770	0.2503	Dunn's	MB299Bshi^{ts1} vs MB299B/+	9	9	-2.0000	>0.9999	ns
						MB299B/shi^{ts1} vs shi^{ts1}/+	9	10	-6.133	0.2092	ns
Fig. 9F	One way ANOVA	2, 45	2.631	0.0831	Sidak's	MB299B/dTrpA1 vs MB299B/+	17	15	-8.711	0.1548	ns
						MB299B/dTrpA1 vs dTrpA1/+	17	16	-10.14	0.0783	ns
Fig. 10D	One way ANOVA	2, 48	0.1022	0.9030	Sidak's	VT064246/Dop1R1^{RNAi} vs Dop1R1^{RNAi}/+	19	14	1.385	0.9814	ns
						VT064246/Dop1R1^{RNAi} vs VT064246/+	19	18	3.379	0.8805	ns
Fig. 10E	One way ANOVA	3, 64	14.31	0.0008	Dunn's	VT064246/Dop1R1^{RNAi} vs Dop1R1^{RNAi}/+	22	22	-20.14	0.0007	***
						VT064246/Dop1R1^{RNAi} vs VT064246/+	22	20	-16.10	0.0102	*
Fig. 10G	One way ANOVA	2, 53	0.5260	0.5940	Sidak's	VT064246/Dop1R2^{RNAi} vs Dop1R2^{RNAi}/+	19	17	-6.487	0.5529	ns
						VT064246/Dop1R2^{RNAi} vs VT064246/+	19	20	-4.730	0.7080	ns
Fig. 10H	One way ANOVA	3, 43	8.302	0.0009	Sidak's	VT064246/Dop1R2^{RNAi} vs Dop1R2^{RNAi}/+	15	15	-18.04	0.0240	*
						VT064246/Dop1R2^{RNAi} vs VT064246/+	15	16	-27.24	0.0005	***
Fig. 11E	One way ANOVA	2, 53	9.050	0.0004	Dunn's	MB310C/dTrpA1 vs MB310C/+	17	20	-23.64	0.0002	****
						MB310C/dTrpA1 vs dTrpA1/+	17	19	-16.37	0.0113	*
Fig. 11F	One way ANOVA	2, 49	11.31	＜0.0001	Sidak's	MB310C/shi^{ts1} vs MB310C/+	17	17	-17.67	0.0045	**
						MB310C/shi^{ts1} vs shi^{ts1}/+	17	18	-25.12	＜0.0001	****
Fig. S1B	One way ANOVA	2, 41	0.04409	0.9569	Sidak's	c316/dTrpA1 vs c316/+	16	15	-1.700	0.9633	ns
						c316/dTrpA1 vs dTrpA1/+	16	13	-1.906	0.9574	ns
Fig. S3A	One way ANOVA	3, 21	15.37	＜0.0001	Dunn's	elav/Dop1R1^{RNAi} vs Dop1R1^{RNAi}/+	9	7	-9.286	0.0060	**
						elav/Dop1R1^{RNAi} vs elav/+	9	5	-12.20	0.0008	***
Fig. S3B	One way ANOVA	3, 19	13.39	＜0.0001	Dunn's	elav/Dop1R2^{RNAi} vs Dop1R2^{RNAi}/+	8	5	-10.30	0.0026	**
						elav/Dop1R2^{RNAi} vs elav/+	8	6	-8.833	0.0073	**
