# Supplementary material for: Brief disruption of activity in a subset of dopaminergic neurons during consolidation impairs long-term memory by fragmenting sleep
Source: eLife. 2026 Sep 23;14:RP104862. doi: 10.7554/eLife.104862 (PMC13600697; doi:10.7554/eLife.104862)
Supplement: Supplementary file 2 [file elife-104862-supp2.pdf]

Table 2. Unpaired t test for 24 h memory (Related to Figs. 6 and 9)

	Comparisions	Test	df	t	p
Fig. 6F	MB299B/dTrpA1: Activation vs No-activation	Unpaired t test	19	2.9940	0.0075
	MB043B/dTrpA1: Activation vs No-activation	Unpaired t test	9	4.7900	0.0010
	MB213B/dTrpA1: Activation vs No-activation	Unpaired t test	11	0.7516	0.4680
	MB025B/dTrpA1: Activation vs No-activation	Unpaired t test	14	0.3025	0.7667
	MB032B/dTrpA1: Activation vs No-activation	Unpaired t test	14	0.3011	0.7677
	MB315C/dTrpA1: Activation vs No-activation	Unpaired t test	22	0.7166	0.4812
Fig. 9G	MB299B/dTrpA1: THIP- vs THIP+	Unpaired t test	32	2.8970	0.0067
