# Supplementary material for: Brief disruption of activity in a subset of dopaminergic neurons during consolidation impairs long-term memory by fragmenting sleep
Source: eLife. 2026 Sep 23;14:RP104862. doi: 10.7554/eLife.104862 (PMC13600697; doi:10.7554/eLife.104862)
Supplement: Supplementary file 3 [file elife-104862-supp3.pdf]

Table 3. Two-way ANOVA for functional imaging and memory tests (Related to Figs. 2, 8, 10 and Fig. 10-figsupp1)

		Group		Time		Interaction		Post-hoc Test	Comparisions		Adjusted p Value	Summary
	DFn, DFd	F	p	F	p	F	p					
Fig. 2A	2, 48	16.20	＜0.0001	48.65	＜0.0001	20.27	＜0.0001	Tukey's	0-30 s	R58E02＞P2X2;VT064246＞GCaMP6f: AHL vs AHL+ATP	0.7409	ns
										AHL vs AHL+ATP: R58E02＞P2X2;VT064246＞GCaMP6f vs +＞P2X2;VT064246＞GCaMP6f	0.9786	ns
										AHL+ATP: R58E02＞P2X2;VT064246＞GCaMP6f vs +＞P2X2;VT064246＞GCaMP6f	0.6585	ns
									31-120 s	R58E02＞P2X2;VT064246＞GCaMP6f: AHL vs AHL+ATP	＜0.0001	****
										AHL vs AHL+ATP: R58E02＞P2X2;VT064246＞GCaMP6f vs +＞P2X2;VT064246＞GCaMP6f	0.5258	ns
										AHL+ATP: R58E02＞P2X2;VT064246＞GCaMP6f vs +＞P2X2;VT064246＞GCaMP6f	＜0.0001	****
	2, 96	21.60	＜0.0001	32.44	＜0.0001	13.52	＜0.0001	Sidak's	AHL	0-30 s vs 31-120 s	0.1909	ns
									AHL+ATP(+＞P2X2;VT064246＞GCaMP6f)	0-30 s vs 31-120 s	0.8484	ns
									AHL+ATP(R58E02＞P2X2;VT064246＞GCaMP6f)	0-30 s vs 31-120 s	<0.0001	****
Fig. 2B	2, 84	13.50	＜0.0001	24.54	＜0.0001	7.868	0.0007	Sidak's	0-30s		0.9999	ns
									31-60 s	VT064246＞GCaMP6f; R58E02＞CsChrimson vs VT064246＞GCaMP6f;+＞CsChrimson	0.0076	****
									61-120 s		＜0.0001	****
	1, 84	24.54	＜0.0001	13.50	＜0.0001	7.868	0.0007	Tukey's	VT064246＞GCaMP6f;R58E02＞CsChrimson	0-30 s vs 31-60 s	0.0012	**
										0-30 s vs 61-120 s	＜0.0001	****
										31-60 s vs 61-120 s	0.0094	**
Fig. 8B	2, 54	15.46	＜0.0001	29.23	＜0.0001	13.87	＜0.0001	Tukey's	0-30 s	MB299B＞P2X2;VT064246＞GCaMP6f: AHL vs AHL+ATP	0.2995	ns
										AHL vs AHL+ATP: MB299B＞P2X2;VT064246＞GCaMP6f vs +＞P2X2;VT064246＞GCaMP6f	0.988	ns
										AHL+ATP: MB299B＞P2X2;VT064246＞GCaMP6f vs +＞P2X2;VT064246＞GCaMP6f	0.5742	ns
									31-120 s	MB299B＞P2X2;VT064246＞GCaMP6f: AHL vs AHL+ATP	＜0.0001	****
										AHL vs AHL+ATP: MB299B＞P2X2;VT064246＞GCaMP6f vs +＞P2X2;VT064246＞GCaMP6f	0.2327	ns
										AHL+ATP: R58E02＞P2X2;VT064246＞GCaMP6f vs +＞P2X2;VT064246＞GCaMP6f	0.0002	***
	2，108	20.82	＜0.0001	19.1	＜0.0001	9.065	0.0002	Sidak's	AHL	0-30 s vs 31-120 s	0.9694	ns
									AHL+ATP(+＞P2X2;VT064246＞GCaMP6f)	0-30 s vs 31-120 s	0.3381	ns
									AHL+ATP(R58E02＞P2X2;VT064246＞GCaMP6f)	0-30 s vs 31-120 s	＜0.0001	****
Fig. 8D	1,174	0.5720	0.4505	1.839	0.1371	1.198	0.2449	Sidak's	Post-training time-1		0.9565	ns
									Post-training time-2		>0.9999	ns
									Post-training time-3		>0.9999	ns
									Post-training time-4		>0.9999	ns
									Post-training time-5		>0.9999	ns
									Post-training time-6		>0.9999	ns
									Post-training time-7		>0.9999	ns
									Post-training time-8		0.9992	ns
									Post-training time-9		>0.9999	ns
									Post-training time-10		>0.9999	ns
									Post-training time-11	Un-trained: PAM-α1 vs DPM	>0.9999	ns
									Post-training time-12		0.9313	ns
									Post-training time-13		>0.9999	ns
									Post-training time-14		0.9975	ns
									Post-training time-15		>0.9999	ns
									Post-training time-16		>0.9999	ns
									Post-training time-17		>0.9999	ns
									Post-training time-18		0.9303	ns
									Post-training time-19		0.9949	ns
									Post-training time-20		0.9903	ns
									Post-training time-21		>0.9999	ns
Fig. 8E	1,171	3.055	0.0823	2.082	0.0032	1.211	0.2342	Sidak's	Post-training time-1		0.9911	ns
									Post-training time-2		>0.9999	ns
									Post-training time-3		0.9998	ns
									Post-training time-4		>0.9999	ns
									Post-training time-5		0.9997	ns
									Post-training time-6		>0.9999	ns
									Post-training time-7		>0.9999	ns

									Post-training time-8		0.6374	ns
									Post-training time-9		>0.9999	ns
									Post-training time-10		>0.9999	ns
									Post-training time-11	Trained: PAM-α1 vs DPM	0.9981	ns
									Post-training time-12		0.9973	ns
									Post-training time-13		0.0857	ns
									Post-training time-14		0.9992	ns
									Post-training time-15		0.9513	ns
									Post-training time-16		0.8802	ns
									Post-training time-17		0.8249	ns
									Post-training time-18		0.6873	ns
									Post-training time-19		0.9718	ns
									Post-training time-20		0.9953	ns
									Post-training time-21		0.9928	ns
Fig. 8F	3, 345	3.177	0.0243	2.907	0.0719	2.955	0.0074	Tukey's		MB299B-No T vs. MB299B-T	0.8525	ns
									post-training time 1 h	MB299B-No T vs. VT064246-No T	0.8387	ns
										MB299B-T vs. VT064246-T	0.4730	ns
										VT064246-No T vs. VT064246-T	0.3546	ns
										MB299B-No T vs. MB299B-T	0.8589	ns
									post-training time 2 h	MB299B-No T vs. VT064246-No T	0.7854	ns
										MB299B-T vs. VT064246-T	0.7890	ns
										VT064246-No T vs. VT064246-T	0.0744	ns
										MB299B-No T vs. MB299B-T	0.9357	ns
									post-training time 3 h	MB299B-No T vs. VT064246-No T	0.6226	ns
										MB299B-T vs. VT064246-T	0.2274	ns
										VT064246-No T vs. VT064246-T	0.0005	***
Fig. 10B	2, 30	4.785	0.0157	90.16	＜0.0001	4.799	0.0155	Dunnett's		wCS vs Dop1R1-KK	0.9998	ns
									0-30 s	wCS vs Dop1R2-GD	0.9999	ns
										Dop1R1-KK vs Dop1R2-GD	0.9994	ns
										wCS vs Dop1R1-KK	0.0209	*
									31-240 s	wCS vs Dop1R2-GD	0.2665	ns
										Dop1R1-KK vs Dop1R2-GD	0.0002	***
Fig. 10C	2, 122	13.99	＜0.0001	2645	＜0.0001	6.955	0.0014	Sidak's	LP(Total sleep)	VT064246/Dop1R1^{RNAi} vs Dop1R1^{RNAi}/+	＜0.0001	****
										VT064246/Dop1R1^{RNAi} vs VT064246/+	＜0.0001	****
									DP(Total sleep)	VT064246/Dop1R1^{RNAi} vs Dop1R1^{RNAi}/+	0.0321	*
										VT064246/Dop1R1^{RNAi} vs VT064246/+	0.3955	ns
	2, 122	8.348	0.0004	66.19	＜0.0001	7.535	0.0008	Dunnett's	LP(# of episodes)	VT064246/Dop1R1^{RNAi} vs Dop1R1^{RNAi}/+	0.5045	ns
										VT064246/Dop1R1^{RNAi} vs VT064246/+	＜0.0001	****
									DP(# of episodes)	VT064246/Dop1R1^{RNAi} vs Dop1R1^{RNAi}/+	0.0060	**
										VT064246/Dop1R1^{RNAi} vs VT064246/+	0.1710	ns
	2, 122	23.59	＜0.0001	1290	＜0.0001	18.66	＜0.0001	Dunnett's	LP(Pwake)	VT064246/Dop1R1^{RNAi} vs Dop1R1^{RNAi}/+	＜0.0001	****
										VT064246/Dop1R1^{RNAi} vs VT064246/+	＜0.0001	****
									DP(Pwake)	VT064246/Dop1R1^{RNAi} vs Dop1R1^{RNAi}/+	0.3129	ns
										VT064246/Dop1R1^{RNAi} vs VT064246/+	0.9947	ns
Fig. 10F	2, 91	0.5364	0.5867	2159	＜0.0001	0.7245	0.4922	Sidak's	LP(Total sleep)	VT064246/Dop1R2^{RNAi} vs Dop1R2^{RNAi}/+	0.9935	ns
										VT064246/Dop1R2^{RNAi} vs VT064246/+	0.9733	ns
									DP(Total sleep)	VT064246/Dop1R2^{RNAi} vs Dop1R2^{RNAi}/+	0.5317	ns
										VT064246/Dop1R2^{RNAi} vs VT064246/+	0.9971	ns
	2, 91	4.098	0.0198	82.19	＜0.0001	0.9814	0.3787	Sidak's	LP(# of episodes)	VT064246/Dop1R2^{RNAi} vs Dop1R2^{RNAi}/+	0.9196	ns
										VT064246/Dop1R2^{RNAi} vs VT064246/+	>0.9999	ns
									DP(# of episodes)	VT064246/Dop1R2^{RNAi} vs Dop1R2^{RNAi}/+	0.0193	*
										VT064246/Dop1R2^{RNAi} vs VT064246/+	0.3626	ns
	2, 91	3.425	0.0368	849.6	＜0.0001	3.982	0.0220	Dunnett's	LP(Pwake)	VT064246/Dop1R2^{RNAi} vs Dop1R2^{RNAi}/+	0.9928	ns
										VT064246/Dop1R2^{RNAi} vs VT064246/+	0.0024	**
									DP(Pwake)	VT064246/Dop1R2^{RNAi} vs Dop1R2^{RNAi}/+	0.6227	ns
										VT064246/Dop1R2^{RNAi} vs VT064246/+	0.7357	ns
Fig10-figsupp1C	1, 23	21,77	0.0001	24.94	＜0.0001	29.45	＜0.0001	Sidak's	0-30 s	AHL+TTX vs AHL+TTX+DA	0.5129	ns
									31-120 s	AHL+TTX vs AHL+TTX+DA	＜0.0001	****
	1, 24	13.02	0.0014	33.45	＜0.0001	17.92	0.0003	Sidak's	AHL+TTX	0-30 s vs 31-120 s	0.9655	ns
									AHL+TTX+DA	0-30 s vs 31-120 s	＜0.0001	****
Fig10_figsupp1D	1, 22	22.04	0.0001	77.04	＜0.0001	26.46	＜0.0001	Sidak's	0-30 s	AHL+TTX vs AHL+TTX+DA	0.2944	ns
									31-120 s	AHL+TTX vs AHL+TTX+DA	＜0.0001	****
	1, 22	26.74	＜0.0001	51.19	＜0.0001	11.18	0.0029	Sidak's	AHL+TTX	0-30 s vs 31-120 s	0.1380	ns
									AHL+TTX+DA	0-30 s vs 31-120 s	＜0.0001	****
Fig10_figsupp1E	1, 40	5.345	0.0260	2.876	＜0.0001	5.899	0.0197	Sidak's	0-30 s	AHL+TTX vs AHL+TTX+DA	0.7618	ns
									31-120 s	AHL+TTX vs AHL+TTX+DA	0.0034	**
	1, 28	7.551	0.0104	2.582	0.1142	2.434	0.1300	Sidak's	AHL+TTX	0-30 s vs 31-120 s	0.8620	ns
									AHL+TTX+DA	0-30 s vs 31-120 s	0.0308	*
