# Supplementary material for: Brief disruption of activity in a subset of dopaminergic neurons during consolidation impairs long-term memory by fragmenting sleep
Source: eLife. 2026 Sep 23;14:RP104862. doi: 10.7554/eLife.104862 (PMC13600697; doi:10.7554/eLife.104862)
Supplement: Supplementary file 4 [file elife-104862-supp4.pdf]

Table 4. Two-way ANOVA for sleep analysis (Related to Figs. 4, 5, 7, 9, 11, S1, S2)

			Group		Time		Interaction	
	Data	DFn, DFd	F	p	F	p	F	p
Fig. 4A	Total sleep	2, 122	10.45	＜0.0001	263.7	＜0.0001	7.493	＜0.0001
	Sleep episodes	2, 122	2.509	0.0856	13.84	＜0.0001	6.577	＜0.0001
	P(wake)	2, 122	12.11	＜0.0001	196.7	＜0.0001	6.504	＜0.0001

Dunnett's multiple comparisons

	R58E02/dTrpA1 vs R58E02/+			R58E02/dTrpA1 vs dTrpA1/+			Summary		
	Total sleep	Sleep episodes	P(wake)	Total sleep	Sleep episodes	P(wake)	Total sleep	Sleep episodes	P(wake)
ZT0-1	0.9756	0.8063	0.9645	0.9806	0.9865	0.0277	ns	ns	ns
ZT1-2	0.2459	0.1827	0.1497	0.9896	0.8710	0.5054	ns	ns	ns
ZT2-3	0.0007	0.0031	0.0008	0.9049	0.9681	0.8836	ns	ns	ns
ZT3-4	0.0004	0.0006	0.0001	0.9980	0.8952	0.6851	ns	ns	ns
ZT4-5	<0.0001	0.2933	<0.0001	0.5611	0.3347	0.3473	ns	ns	ns
ZT5-6	0.1148	0.0319	0.3845	0.0009	<0.0001	0.0111	ns	*	ns
ZT6-7	<0.0001	0.4502	<0.0001	<0.0001	0.0048	0.0052	****	ns	**
ZT7-8	<0.0001	0.0182	<0.0001	<0.0001	0.0010	0.0017	****	*	**
ZT8-9	<0.0001	0.0018	<0.0001	<0.0001	0.0016	<0.0001	****	**	****
ZT9-10	<0.0001	0.0036	0.0002	0.0026	0.1240	0.0026	**	ns	**
ZT10-11	0.0038	0.0090	<0.0001	0.0677	0.0406	0.0133	ns	*	*
ZT11-12	0.028	0.0108	0.0377	0.9890	0.9447	0.5754	ns	ns	ns
ZT12-13	0.9747	0.1524	0.7279	0.9914	0.0032	0.7997	ns	ns	ns
ZT13-14	0.9163	0.0007	0.7183	0.8430	0.0080	0.9813	ns	**	ns
ZT14-15	0.6078	<0.0001	0.8074	0.0909	0.0100	0.2094	ns	*	ns
ZT15-16	0.3992	<0.0001	0.2807	0.0159	0.1387	0.0447	ns	ns	ns
ZT16-17	0.7681	<0.0001	0.9273	0.0006	0.0019	0.0011	ns	**	ns
ZT17-18	0.2873	0.0029	0.2764	0.0279	0.0041	0.0005	ns	**	ns
ZT18-19	>0.9999	<0.0001	0.1640	0.5784	0.0019	0.1675	ns	**	ns
ZT19-20	0.9898	0.0060	0.8583	0.4328	0.0124	0.2425	ns	*	ns
ZT20-21	0.8351	0.0123	0.9942	0.8891	0.1945	0.8824	ns	ns	ns
ZT21-22	0.4996	0.0129	0.9399	0.7758	0.2699	0.7315	ns	ns	ns
ZT22-23	0.7468	0.0010	0.9476	0.3676	0.1273	0.2538	ns	ns	ns
ZT23-24	0.5168	0.0728	0.2801	0.3308	0.0447	0.9372	ns	ns	ns
ZT24-25	0.3769	0.6626	0.9080	0.1401	0.4485	0.2559	ns	ns	ns
ZT25-26	0.1607	0.1756	0.2289	0.9114	0.5869	0.8935	ns	ns	ns
ZT26-27	0.2468	0.0393	0.0542	0.6703	0.6408	0.8712	ns	ns	ns
ZT27-28	0.6703	0.1063	0.0501	0.0053	0.1016	0.6792	ns	ns	ns
ZT28-29	0.1917	0.7103	0.0849	0.0345	0.7491	0.0855	ns	ns	ns

			Group		Time		Interaction	
	Data	DFn, DFd	F	p	F	p	F	p
Fig. 4B	Total sleep	2, 104	9.135	0.0002	228.7	＜0.0001	5.274	＜0.0001
	Sleep episodes	2, 104	11.32	＜0.0001	21.39	＜0.0001	4.321	＜0.0001
	P(wake)	2, 104	8.225	0.0005	178.2	＜0.0001	5.579	＜0.0001

Dunnett's multiple comparisons

	R58E024/shi^{ts1} vs R58E02/+			R58E02/shi^{ts1} vs shi^{ts1}/+			Summary		
	Total sleep	Sleep episodes	P(wake)	Total sleep	Sleep episodes	P(wake)	Total sleep	Sleep episodes	P(wake)
ZT0-1	0.0181	0.0094	-	0.8051	0.9767	-	ns	ns	-
ZT1-2	<0.0001	<0.0001	<0.0001	0.3105	0.6616	0.9997	ns	ns	ns
ZT2-3	0.0079	0.0022	0.0014	0.9927	0.6353	0.8757	ns	ns	ns
ZT3-4	0.0250	0.0310	0.0027	0.8589	0.5669	0.9968	ns	ns	ns
ZT4-5	0.0084	0.1151	0.0043	0.2469	0.6219	0.8930	ns	ns	ns
ZT5-6	0.0163	0.7762	0.0018	0.2830	0.6890	0.1746	ns	ns	ns
ZT6-7	0.0001	0.1505	0.0027	0.0010	0.4891	0.1983	**	ns	ns
ZT7-8	0.6738	0.4714	0.0147	0.0012	0.0017	0.0526	ns	ns	ns
ZT8-9	0.9676	0.6733	0.8487	0.3096	0.4022	0.1828	ns	ns	ns
ZT9-10	0.8522	0.2422	0.3108	0.0011	0.9952	0.6962	ns	ns	ns
ZT10-11	0.9966	0.8254	0.0683	0.0039	0.8336	<0.0001	ns	ns	ns
ZT11-12	0.0268	0.2804	0.0678	0.9538	0.2620	0.8916	ns	ns	ns
ZT12-13	0.2144	0.3250	0.8537	0.0083	0.0513	0.1085	ns	ns	ns
ZT13-14	0.8022	0.5470	0.9997	0.7276	0.9974	0.9802	ns	ns	ns
ZT14-15	0.0108	0.0149	0.0022	0.0005	0.5129	0.0040	*	ns	**
ZT15-16	0.0470	<0.0001	0.2140	0.0015	0.0002	0.0010	*	***	ns
ZT16-17	0.0093	0.0029	0.0174	0.0003	0.0004	0.0009	**	**	*
ZT17-18	0.1503	0.0012	0.0009	0.0008	0.0031	<0.0001	ns	**	***

ZT18-19	0.3069	0.0002	0.2621	0.0013	<0.0001	0.0012	ns	***	ns
ZT19-20	0.7608	0.0005	0.4389	<0.0001	0.0182	0.0005	ns	*	ns
ZT20-21	0.9067	0.0016	0.1881	0.0002	<0.0001	0.0001	ns	**	ns
ZT21-22	0.1312	0.0174	0.3207	0.0004	0.0008	0.0008	ns	*	ns
ZT22-23	0.7829	0.4124	0.3113	0.0014	0.0055	0.0031	ns	ns	ns
ZT23-24	0.9600	0.1246	0.6941	0.0003	0.0081	0.0009	ns	ns	ns
ZT24-25	0.5007	0.5953	0.4108	0.0642	0.0241	0.0114	ns	ns	ns
ZT25-26	0.1666	0.3557	0.6301	0.3275	0.2272	0.0052	ns	ns	ns
ZT26-27	0.1584	0.2923	0.0050	0.7385	0.9887	0.9384	ns	ns	ns
ZT27-28	0.5233	0.5943	0.5007	0.5311	0.6781	0.6761	ns	ns	ns
ZT28-29	0.7872	0.1363	0.7345	0.0305	0.4297	0.2494	ns	ns	ns
ZT29-30	0.4707	0.4440	0.9097	<0.0001	0.9572	0.0419	ns	ns	ns
ZT30-31	0.4530	0.4523	0.2749	0.0001	0.0805	<0.0001	ns	ns	ns

			Group		Time		Interaction	
	Data	DFn, DFd	F	p	F	p	F	p
Fig. 4C	Total sleep	2, 113	21.95	<0.0001	346.6	<0.0001	16.94	<0.0001
	Sleep episodes	2, 113	38.34	<0.0001	13.33	<0.0001	5.525	<0.0001
	P(wake)	2, 112	7.799	0.0007	247.6	<0.0001	11.82	<0.0001

Dunnett's multiple comparisons

	c316/shi^{ts1} vs c316/+			c316/shi^{ts1} vs shi^{ts1}/+			Summary		
	Total sleep	Sleep episodes	P(wake)	Total sleep	Sleep episodes	P(wake)	Total sleep	Sleep episodes	P(wake)
ZT0-1	0.1048	0.1433	0.8009	0.0053	0.0490	0.1680	ns	ns	ns
ZT1-2	0.1867	0.1312	0.0051	0.1948	0.1097	0.0001	ns	ns	**
ZT2-3	0.1621	0.1417	0.2292	0.2487	0.8214	0.0791	ns	ns	ns
ZT3-4	0.1985	0.1068	0.2110	0.6183	0.9873	0.7170	ns	ns	ns
ZT4-5	0.7876	0.0814	0.9244	0.0131	0.9763	0.0451	ns	ns	ns
ZT5-6	0.0374	0.0008	0.9977	0.9417	0.0011	0.0011	ns	**	ns
ZT6-7	<0.0001	<0.0001	<0.0001	0.5373	0.2511	0.0134	ns	ns	*
ZT7-8	0.0244	0.1735	<0.0001	0.0637	0.1970	0.5041	ns	ns	ns
ZT8-9	0.9933	0.0032	0.5954	<0.0001	0.0703	0.0461	ns	ns	ns
ZT9-10	0.5792	0.7133	0.9481	<0.0001	0.0308	<0.0001	ns	ns	ns
ZT10-11	0.9478	0.5248	0.8991	<0.0001	0.9233	<0.0001	ns	ns	ns
ZT11-12	0.0028	0.0011	0.0042	0.7002	0.1342	0.1334	ns	ns	ns
ZT12-13	0.1545	0.3935	0.1160	0.0986	0.2958	0.8508	ns	ns	ns
ZT13-14	0.0174	0.0229	0.1881	0.0002	0.2108	0.0232	*	ns	ns
ZT14-15	0.0002	<0.0001	<0.0001	<0.0001	0.0002	<0.0001	***	***	****
ZT15-16	<0.0001	<0.0001	<0.0001	0.0003	0.0031	0.0004	***	**	***
ZT16-17	0.0002	<0.0001	<0.0001	0.0001	<0.0001	0.0001	***	****	***
ZT17-18	<0.0001	<0.0001	<0.0001	0.0008	0.0169	0.0001	***	*	***
ZT18-19	<0.0001	<0.0001	<0.0001	0.0003	0.0001	0.0007	***	***	***
ZT19-20	<0.0001	<0.0001	<0.0001	0.0007	0.0011	0.0009	***	**	***
ZT20-21	<0.0001	<0.0001	<0.0001	0.0001	0.0016	0.0003	***	**	***
ZT21-22	0.0137	0.0010	0.0005	0.0055	0.0032	0.0011	*	**	**
ZT22-23	0.0045	0.0118	0.0711	<0.0001	0.0008	<0.0001	**	*	ns
ZT23-24	0.0127	0.0011	0.0025	0.0002	0.0005	0.0003	*	**	**
ZT24-25	0.8349	0.3781	0.1102	0.3149	0.3449	0.0019	ns	ns	ns
ZT25-26	0.0147	0.0113	0.0099	0.5632	0.0657	0.0407	ns	ns	*
ZT26-27	<0.0001	0.0001	0.0386	0.6998	0.0850	0.3568	ns	ns	ns
ZT27-28	0.0095	0.0964	0.0063	0.2883	0.8494	0.9278	ns	ns	ns
ZT28-29	0.0003	0.0028	0.0061	0.0001	0.9295	0.0663	***	ns	ns

			Group		Time		Interaction	
	Data	DFn, DFd	F	p	F	p	F	p
Fig. 4D	Total sleep	2, 63	12.78	<0.0001	104.1	<0.0001	6.416	<0.0001
	Sleep episodes	2, 63	11.13	<0.0001	8.574	<0.0001	5.329	<0.0001
	P(wake)	2, 56	3.284	0.0448	68.15	<0.0001	5.795	<0.0001

Dunnett's multiple comparisons

	VT064246/shi^{ts1} vs VT064246/+			VT064246/shi^{ts1}vs shi^{ts1}/+			Summary		
	Total sleep	Sleep episodes	P(wake)	Total sleep	Sleep episodes	P(wake)	Total sleep	Sleep episodes	P(wake)
ZT0-1	0.6014	0.1033	0.8663	0.1096	0.1622	0.2061	ns	ns	ns
ZT1-2	0.8298	0.0350	0.8296	0.1747	0.9427	0.1743	ns	ns	ns
ZT2-3	0.5753	0.1187	0.9827	0.4390	0.8938	0.1038	ns	ns	ns
ZT3-4	0.0919	0.0350	0.4160	>0.9999	0.1342	0.4869	ns	ns	ns
ZT4-5	0.0232	0.9933	0.6256	0.0151	0.7952	0.9207	*	ns	ns

ZT5-6	0.8659	0.9558	0.3036	0.2953	0.6880	0.5350	ns	ns	ns
ZT6-7	<0.0001	0.6634	0.0008	0.2530	0.2875	0.5392	ns	ns	ns
ZT7-8	0.4548	0.4910	0.0004	0.0632	0.2992	0.6562	ns	ns	ns
ZT8-9	0.0003	0.7733	0.2305	0.0014	0.4441	0.2841	**	ns	ns
ZT9-10	<0.0001	0.0052	0.0122	0.0002	0.3562	0.0101	***	ns	*
ZT10-11	<0.0001	<0.0001	0.0258	0.2955	0.5178	0.4076	ns	ns	ns
ZT11-12	<0.0001	0.0002	0.0683	0.5733	0.9356	0.9960	ns	ns	ns
ZT12-13	0.0002	0.0662	0.1071	0.2021	0.8383	0.6134	ns	ns	ns
ZT13-14	0.0058	0.1391	0.0659	0.0515	0.1148	0.4398	ns	ns	ns
ZT14-15	0.0020	0.1087	0.1218	0.0104	0.1525	0.2061	*	ns	ns
ZT15-16	0.0008	0.0056	0.0835	0.0071	0.0106	0.0883	**	*	ns
ZT16-17	0.0035	0.0813	0.1588	0.0617	0.0130	0.1196	ns	ns	ns
ZT17-18	0.0012	0.0392	0.1032	0.0093	0.0781	0.1483	**	ns	ns
ZT18-19	0.0215	0.0525	0.2687	0.0325	0.0466	0.0915	*	ns	ns
ZT19-20	0.0395	0.1104	0.2266	0.1014	0.0598	0.1230	ns	ns	ns
ZT20-21	0.1632	0.0208	0.4819	0.1634	0.0194	0.2061	ns	*	ns
ZT21-22	0.1451	0.1005	0.7523	0.6687	0.0731	0.4734	ns	ns	ns
ZT22-23	0.6272	0.1125	0.4157	0.9994	0.4994	0.4309	ns	ns	ns
ZT23-24	0.0636	0.0111	0.5989	0.4181	0.0054	0.2601	ns	*	ns
ZT24-25	0.0007	0.0004	0.2471	0.7169	0.6568	0.2810	ns	ns	ns
ZT25-26	0.0005	0.0002	0.3645	0.5520	0.6667	0.2427	ns	ns	ns
ZT26-27	0.0031	0.0326	0.3271	0.9418	0.5021	0.5079	ns	ns	ns
ZT27-28	0.0045	0.0179	0.3211	0.4364	0.6784	0.5074	ns	ns	ns
ZT28-29	0.0044	0.0946	0.3225	0.6624	0.9991	0.2044	ns	ns	ns

			Group		Time		Interaction	
	Data	DFn, DFd	F	p	F	p	F	p
Fig. 5A	Total sleep	2, 120	32.18	＜0.0001	343.8	＜0.0001	12.58	＜0.0001
	Sleep episodes	2, 120	27.39	＜0.0001	16.31	＜0.0001	5.367	＜0.0001
	P(wake)	2, 120	39.01	＜0.0001	264.0	＜0.0001	11.71	＜0.0001

Dunnett's multiple comparisons

	R58E02/dTrpA1 vs R58E02/+			R58E02/dTrpA1 vs dTrpA1/+			Summary		
	Total sleep	Sleep episodes	P(wake)	Total sleep	Sleep episodes	P(wake)	Total sleep	Sleep episodes	P(wake)
ZT0-1	0.9086	0.8154	0.5343	0.9342	0.9490	0.6511	ns	ns	ns
ZT1-2	0.0383	0.0006	0.0511	0.8805	0.2848	0.9609	ns	ns	ns
ZT2-3	<0.0001	<0.0001	<0.0001	0.9143	0.9886	0.9971	ns	ns	ns
ZT3-4	<0.0001	0.0097	<0.0001	0.9898	0.7700	0.6867	ns	ns	ns
ZT4-5	<0.0001	0.0165	<0.0001	0.3169	0.5500	0.9712	ns	ns	ns
ZT5-6	<0.0001	0.9966	<0.0001	0.0024	0.9747	0.0659	**	ns	ns
ZT6-7	<0.0001	<0.0001	<0.0001	<0.0001	0.0010	0.0004	****	***	***
ZT7-8	<0.0001	<0.0001	<0.0001	＞0.9999	0.8920	0.9271	ns	ns	ns
ZT8-9	<0.0001	<0.0001	<0.0001	0.4656	0.7701	0.9989	ns	ns	ns
ZT9-10	<0.0001	<0.0001	<0.0001	0.9999	0.9275	0.7883	ns	ns	ns
ZT10-11	0.0072	0.0086	<0.0001	0.4619	0.5395	0.7640	ns	ns	ns
ZT11-12	0.2348	0.0630	0.6876	0.0365	0.0433	0.1069	ns	ns	ns
ZT12-13	<0.0001	0.0010	0.0003	0.0001	0.0050	0.3617	***	**	ns
ZT13-14	<0.0001	0.4330	<0.0001	0.0005	0.9703	0.0067	***	ns	**
ZT14-15	0.0019	0.1080	0.0003	0.0004	0.0718	0.0001	**	ns	***
ZT15-16	0.0058	0.2221	0.0081	0.0014	0.7349	0.0084	**	ns	**
ZT16-17	0.0151	0.7221	0.0038	0.0155	0.2442	0.0349	*	ns	*
ZT17-18	0.5671	0.9007	0.5788	0.4124	0.9989	0.3067	ns	ns	ns
ZT18-19	0.9169	0.6717	0.4843	0.6928	0.8824	0.8054	ns	ns	ns
ZT19-20	0.5228	0.7593	0.9883	0.3200	0.8915	0.0783	ns	ns	ns
ZT20-21	0.2034	0.0700	0.6126	0.9719	0.3042	0.8387	ns	ns	ns
ZT21-22	0.0373	0.9963	0.0364	0.4777	0.7408	0.2915	ns	ns	ns
ZT22-23	0.2897	0.9932	0.0325	0.9445	0.6610	0.3055	ns	ns	ns
ZT23-24	0.8547	0.6399	0.8373	0.6993	0.5527	0.5578	ns	ns	ns
ZT24-25	0.0827	0.1046	0.7116	0.1276	0.4522	0.6013	ns	ns	ns
ZT25-26	0.0188	0.0101	0.2142	0.8966	0.8637	0.9667	ns	ns	ns
ZT26-27	<0.0001	0.0001	<0.0001	0.9692	0.9879	0.7914	ns	ns	ns
ZT27-28	<0.0001	0.0006	<0.0001	0.7390	0.7811	0.9991	ns	ns	ns
ZT28-29	<0.0001	0.1204	<0.0001	0.2086	0.4445	0.1619	ns	ns	ns

			Group		Time		Interaction	
	Data	DFn, DFd	F	p	F	p	F	p
	Total sleep	2, 85	0.8538	0.4294	145.1	＜0.0001	2.800	＜0.0001

	Data	DFn, DFd	F	p	F	p	F	p
Fig. 5B	Sleep episodes	2, 85	10.55	<0.0001	16.12	<0.0001	1.593	<0.0001
	P(wake)	2, 85	0.1322	0.8764	122.3	<0.0001	3.910	<0.0001

Dunnett's multiple comparisons

	R58E02/shi^{ts1} vs R58E02/+			R58E02/shi^{ts1} vs shi^{ts1}/+			Summary		
	Total sleep	Sleep episodes	P(wake)	Total sleep	Sleep episodes	P(wake)	Total sleep	Sleep episodes	P(wake)
ZT0-1	0.8005	0.2826	0.0435	0.4019	0.8339	0.0771	ns	ns	ns
ZT1-2	0.9382	0.1464	0.0793	0.1645	0.4917	0.1169	ns	ns	ns
ZT2-3	0.1552	0.0021	0.3853	0.7564	0.1270	0.7221	ns	ns	ns
ZT3-4	0.3085	0.1483	0.1580	0.3739	0.5286	0.5679	ns	ns	ns
ZT4-5	0.0856	0.4209	0.1641	0.0247	0.7966	0.0538	ns	ns	ns
ZT5-6	0.7124	0.6537	0.2077	0.2381	0.7568	0.0337	ns	ns	ns
ZT6-7	0.2565	0.4993	0.8119	0.0179	0.9113	0.1739	ns	ns	ns
ZT7-8	0.9895	0.9558	0.2751	0.7794	0.1494	0.0590	ns	ns	ns
ZT8-9	0.9689	0.9665	0.9934	0.9968	0.9880	0.9565	ns	ns	ns
ZT9-10	0.0042	>0.9999	0.609	0.4358	0.8578	0.7616	ns	ns	ns
ZT10-11	0.0331	0.5824	0.0017	0.3377	0.1917	0.5663	ns	ns	ns
ZT11-12	0.9911	0.0647	0.5694	0.3057	0.7984	0.0155	ns	ns	ns
ZT12-13	0.2987	0.9709	0.7210	0.2382	0.6114	0.1945	ns	ns	ns
ZT13-14	0.5461	0.8163	0.6356	0.3262	0.2385	0.9144	ns	ns	ns
ZT14-15	0.4584	0.8238	0.3040	0.2050	0.0029	0.4159	ns	ns	ns
ZT15-16	0.6002	0.9381	0.7440	0.1588	0.0311	0.3794	ns	ns	ns
ZT16-17	0.8609	0.4353	0.3110	0.0467	0.0002	0.0432	ns	ns	ns
ZT17-18	0.3560	0.2718	0.3548	0.0095	0.0114	0.0272	ns	ns	ns
ZT18-19	0.9652	0.3089	0.2594	0.0750	0.0064	0.0130	ns	ns	ns
ZT19-20	0.6333	0.8799	0.6772	0.4020	0.1600	0.1175	ns	ns	ns
ZT20-21	0.5231	0.8895	0.5613	0.3095	0.1607	0.9982	ns	ns	ns
ZT21-22	0.0171	0.9988	0.1173	0.4731	0.0465	0.3027	ns	ns	ns
ZT22-23	0.1985	0.6118	0.0365	0.2963	0.1331	0.8238	ns	ns	ns
ZT23-24	0.9784	0.3963	0.8772	0.4226	0.0608	0.3506	ns	ns	ns
ZT24-25	0.3422	0.7435	0.8284	0.1850	0.1291	0.5364	ns	ns	ns
ZT25-26	0.7121	0.9965	0.8877	0.0971	0.0168	0.0378	ns	ns	ns
ZT26-27	0.1224	0.4326	0.6606	0.1271	0.9026	0.2283	ns	ns	ns
ZT27-28	0.6677	0.0765	0.6609	0.9972	0.1588	0.9780	ns	ns	ns
ZT28-29	0.1900	0.2959	0.2459	0.9980	0.9821	0.7576	ns	ns	ns
ZT29-30	0.8075	0.2680	0.3006	0.9481	0.5323	0.9075	ns	ns	ns
ZT30-31	0.2392	0.2211	0.2012	0.9997	0.9988	0.9419	ns	ns	ns

			Group		Time		Interaction	
	Data	DFn, DFd	F	p	F	p	F	p
Fig. 5C	Total sleep	2, 109	40.01	<0.0001	305.9	<0.0001	12.03	<0.0001
	Sleep episodes	2, 109	17.56	<0.0001	27.15	<0.0001	5.9780	<0.0001
	P(wake)	2, 109	32.74	<0.0001	252.9	<0.0001	14.91	<0.0001

Dunnett's multiple comparisons

	c316/shi^{ts1} vs c316/+			c316/shi^{ts1} vs shi^{ts1}/+			Summary		
	Total sleep	Sleep episodes	P(wake)	Total sleep	Sleep episodes	P(wake)	Total sleep	Sleep episodes	P(wake)
ZT0-1	0.2363	0.0786	0.1531	0.0893	0.0645	0.0042	ns	ns	ns
ZT1-2	0.0073	0.0160	0.0121	0.6278	0.4555	0.0622	ns	ns	ns
ZT2-3	0.0030	0.0012	0.0001	0.8066	0.8262	0.6792	ns	ns	ns
ZT3-4	0.0025	0.0049	<0.0001	0.0003	0.0889	0.0111	**	ns	*
ZT4-5	0.0068	0.2228	<0.0001	<0.0001	0.0416	<0.0001	**	ns	****
ZT5-6	0.0059	0.2774	0.0203	0.0015	0.0047	0.0003	**	ns	*
ZT6-7	0.0004	0.1944	<0.0001	0.1674	0.9849	0.1763	ns	ns	ns
ZT7-8	0.6153	0.5574	0.0191	0.9404	0.9968	0.1813	ns	ns	ns
ZT8-9	0.0221	0.4296	0.4767	0.0083	0.0001	0.2909	*	ns	ns
ZT9-10	0.1397	0.1204	0.0040	<0.0001	<0.0001	<0.0001	ns	ns	**
ZT10-11	0.0312	0.0256	0.0018	0.0004	0.0200	<0.0001	*	*	**
ZT11-12	0.6914	0.7137	0.0019	0.9579	0.9744	0.0140	ns	ns	*
ZT12-13	0.0187	0.1381	0.4596	<0.0001	0.0398	0.0417	*	ns	ns
ZT13-14	0.0414	0.3252	0.0279	<0.0001	0.0304	<0.0001	*	ns	*
ZT14-15	0.7500	0.1517	0.2397	<0.0001	0.0004	0.0010	ns	ns	ns
ZT15-16	0.7791	0.7682	0.9804	0.0004	0.0156	0.0017	ns	ns	ns
ZT16-17	0.4514	0.3751	0.6402	<0.0001	<0.0001	0.0002	ns	ns	ns
ZT17-18	0.0528	0.1956	0.1104	<0.0001	0.0012	<0.0001	ns	ns	ns
ZT18-19	0.1888	0.0408	0.5773	0.0016	0.0019	0.0029	ns	*	ns
ZT19-20	0.5402	0.5251	0.2536	0.0100	0.0078	0.0151	ns	ns	ns

ZT20-21	0.2873	0.1806	0.9350	0.1306	0.1345	0.0418	ns	ns	ns
ZT21-22	0.6358	0.4027	0.2554	0.0108	0.1027	0.0351	ns	ns	ns
ZT22-23	0.1032	0.1278	0.6022	0.0018	0.0143	0.0180	ns	ns	ns
ZT23-24	0.0353	0.1643	0.3655	0.0212	0.1027	0.0361	*	ns	ns
ZT24-25	0.1111	0.0691	0.0423	0.1887	0.2912	0.0167	ns	ns	*
ZT25-26	0.0023	0.0008	0.0002	0.0213	0.0116	0.0066	*	*	**
ZT26-27	0.0037	0.0059	0.0003	＞0.9999	0.9432	0.0291	ns	ns	*
ZT27-28	0.0028	0.0587	<0.0001	0.0008	0.3171	0.1213	**	ns	ns
ZT28-29	0.0002	0.0093	<0.0001	0.0105	0.8054	0.0022	*	ns	ns

			Group		Time		Interaction	
	Data	DFn, DFd	F	p	F	p	F	p
Fig. 5D	Total sleep	2, 120	10.69	＜0.0001	366.0	＜0.0001	3.9710	＜0.0001
	Sleep episodes	2, 120	1.6140	0.2034	32.36	＜0.0001	2.8770	＜0.0001
	P(wake)	2, 108	9.6600	0.0001	275.7	＜0.0001	2.8450	＜0.0001

Dunnett's multiple comparisons

	c316/dTrpA1 vs c316/+			c316/dTrpA1 vs dTrpA1/+			Summary		
	Total sleep	Sleep episodes	P(wake)	Total sleep	Sleep episodes	P(wake)	Total sleep	Sleep episodes	P(wake)
ZT0-1	0.3626	0.0391	0.8978	0.9918	＞0.9999	0.0111	ns	ns	ns
ZT1-2	0.9545	0.9799	0.9973	0.5452	0.6784	0.1896	ns	ns	ns
ZT2-3	0.9474	0.9727	0.0966	0.4805	0.6537	0.9961	ns	ns	ns
ZT3-4	0.9316	0.8585	0.9857	0.1698	0.9464	0.0398	ns	ns	ns
ZT4-5	0.4767	0.9982	0.9864	0.0028	0.3100	0.0016	ns	ns	ns
ZT5-6	0.2708	0.6247	0.8270	0.0008	0.4900	0.0022	ns	ns	ns
ZT6-7	0.4385	0.9366	0.7885	＜0.0001	0.9876	0.0631	ns	ns	ns
ZT7-8	0.0021	0.0587	0.0113	0.0257	0.0587	0.077	*	ns	ns
ZT8-9	0.5146	0.4169	0.8064	0.2170	0.5753	0.3378	ns	ns	ns
ZT9-10	0.0518	0.0213	0.0871	0.7524	0.9972	0.8517	ns	ns	ns
ZT10-11	0.0626	0.0877	0.0092	0.7672	0.7835	0.9346	ns	ns	ns
ZT11-12	0.0121	0.1292	0.7628	0.9415	0.7839	0.2535	ns	ns	ns
ZT12-13	0.0125	0.0010	0.0116	0.0118	0.0097	0.0027	*	**	*
ZT13-14	0.1297	0.3798	0.2562	＜0.0001	0.2680	0.0246	ns	ns	ns
ZT14-15	0.9200	0.9719	0.1479	＜0.0001	0.0002	0.0124	ns	ns	ns
ZT15-16	0.7227	0.6478	0.0932	0.0046	0.0021	0.9846	ns	ns	ns
ZT16-17	0.9513	0.2125	0.3510	0.0581	0.0258	0.9999	ns	ns	ns
ZT17-18	0.4718	0.2965	0.1772	0.9854	0.3905	0.8107	ns	ns	ns
ZT18-19	0.4065	0.9803	0.1455	0.5511	0.0580	0.6864	ns	ns	ns
ZT19-20	0.2573	0.1721	0.8705	0.6872	0.3212	0.6427	ns	ns	ns
ZT20-21	0.9307	0.9430	0.8212	0.9755	0.5198	0.8757	ns	ns	ns
ZT21-22	0.9996	0.8567	0.9889	0.9853	0.8890	0.9376	ns	ns	ns
ZT22-23	0.6563	0.7556	0.8949	0.9631	0.9708	0.8445	ns	ns	ns
ZT23-24	0.9722	0.8131	0.8383	0.9392	0.9815	0.9057	ns	ns	ns
ZT24-25	0.3406	0.3063	0.2709	0.9494	0.9450	0.0613	ns	ns	ns
ZT25-26	0.8390	0.9893	0.4518	0.4362	0.2941	0.1113	ns	ns	ns
ZT26-27	0.9759	0.8060	0.9634	0.1469	0.1217	0.0861	ns	ns	ns
ZT27-28	0.9128	0.7156	0.5508	0.0284	0.1679	0.001	ns	ns	ns
ZT28-29	0.9916	0.6140	0.9656	0.0003	0.0173	0.0050	ns	ns	ns
ZT29-30	0.2575	0.6352	0.7387	0.0025	0.8587	0.0085	ns	ns	ns
ZT30-31	0.9980	0.7240	0.9954	0.0505	0.4597	0.0175	ns	ns	ns

			Group		Time		Interaction	
	Data	DFn, DFd	F	p	F	p	F	p
Fig. 7H	Total sleep	2, 76	8.388	0.0005	169.2	＜0.0001	3.7640	＜0.0001
	Sleep episodes	2, 76	4.602	0.0130	17.64	＜0.0001	2.9960	＜0.0001
	P(wake)	2, 76	3.148	0.0486	131.5	＜0.0001	2.7130	＜0.0001

Dunnett's multiple comparisons

	MB299B/dTrpA1 vs MB299B/+			MB299B/dTrpA1 vs dTrpA1/+			Summary		
	Total sleep	Sleep episodes	P(wake)	Total sleep	Sleep episodes	P(wake)	Total sleep	Sleep episodes	P(wake)
ZT0-1	0.9008	0.7471	-	0.3210	0.9972	-	ns	ns	-
ZT1-2	0.1209	0.0056	0.0438	0.8648	＞0.9999	0.6962	ns	ns	ns
ZT2-3	0.4323	0.2669	0.3740	0.9895	0.9957	0.8727	ns	ns	ns
ZT3-4	0.9515	0.3079	0.8372	0.9240	0.9397	0.9880	ns	ns	ns
ZT4-5	0.0994	0.5627	0.9481	0.0015	0.3609	0.9998	ns	ns	ns
ZT5-6	0.1848	0.5773	0.3472	＜0.0001	0.1328	0.0002	ns	ns	ns
ZT6-7	0.0675	0.0338	0.2039	＜0.0001	0.0343	＜0.0001	ns	*	ns

ZT7-8	0.1650	0.9985	0.6571	0.1190	0.2494	＜0.0001	ns	ns	ns
ZT8-9	0.1921	0.3105	0.9855	0.0015	0.1149	0.0515	ns	ns	ns
ZT9-10	0.4766	0.1086	0.6598	0.8071	0.9734	0.9204	ns	ns	ns
ZT10-11	0.9234	0.8170	0.8971	0.4374	0.1497	0.9152	ns	ns	ns
ZT11-12	0.7611	0.9832	0.8104	0.4730	0.3304	0.6678	ns	ns	ns
ZT12-13	0.4141	0.3131	0.3499	0.9966	0.5251	0.9479	ns	ns	ns
ZT13-14	0.1063	0.1120	0.2001	0.0129	0.0788	0.0673	ns	ns	ns
ZT14-15	0.8599	0.3156	0.3850	0.0029	0.0075	0.0057	ns	ns	ns
ZT15-16	0.9441	0.0110	0.9094	0.0010	0.0010	0.0083	ns	*	ns
ZT16-17	0.6404	0.0068	0.7756	0.0020	0.0001	0.0075	ns	**	ns
ZT17-18	0.0416	0.0037	0.0687	0.0002	＜0.0001	＜0.0001	*	**	ns
ZT18-19	0.0226	0.6235	0.4094	0.0004	0.0149	0.0097	*	ns	ns
ZT19-20	0.0010	0.0007	0.0055	0.0008	＜0.0001	0.0009	***	***	**
ZT20-21	0.0016	0.0003	0.0020	0.0458	＜0.0001	0.0098	*	***	**
ZT21-22	0.0097	0.0198	0.0025	0.0188	0.0016	0.0356	*	*	*
ZT22-23	0.0584	0.0050	0.0526	0.0412	0.0375	0.1694	ns	*	ns
ZT23-24	0.1141	0.7781	0.3733	0.1519	0.1133	0.1542	ns	ns	ns
ZT24-25	0.6262	0.8891	0.9961	0.7042	0.9609	0.5279	ns	ns	ns
ZT25-26	0.5513	0.8899	0.6711	0.4102	0.3740	0.9370	ns	ns	ns
ZT26-27	0.7146	0.9669	0.9096	0.7726	0.7684	0.9991	ns	ns	ns
ZT27-28	0.2084	0.2886	0.1680	0.9489	0.4794	0.9659	ns	ns	ns
ZT28-29	0.1365	0.3233	0.5605	0.4382	0.8724	0.9521	ns	ns	ns
ZT29-30	0.8415	0.9937	0.4147	0.1906	0.9938	0.3155	ns	ns	ns
ZT30-31	0.4714	0.8791	0.7649	0.0561	0.9025	0.4026	ns	ns	ns

			Group		Time		Interaction	
	Data	DFn, DFd	F	p	F	p	F	p
Fig. 7I	Total sleep	2, 103	2.4680	0.0897	294.4	＜0.0001	9.8710	＜0.0001
	Sleep episodes	2, 103	8.9860	0.0003	19.13	＜0.0001	3.5190	＜0.0001
	P(wake)	2, 103	1.3280	0.2696	213.7	＜0.0001	9.2830	＜0.0001

Dunnett's multiple comparisons

	MB299B/shi^{ts1} vs MB299B/+			MB299B/shi^{ts1} vs shi^{ts1}/+			Summary		
	Total sleep	Sleep episodes	P(wake)	Total sleep	Sleep episodes	P(wake)	Total sleep	Sleep episodes	P(wake)
ZT0-1	0.5453	0.9967	0.9595	0.0046	0.1744	0.0008	ns	ns	ns
ZT1-2	0.9548	0.9576	0.9559	0.4535	0.2436	0.0031	ns	ns	ns
ZT2-3	0.8636	0.9626	0.0245	0.9724	0.9883	0.6837	ns	ns	ns
ZT3-4	0.8184	0.9767	0.3053	0.8970	0.9319	0.7827	ns	ns	ns
ZT4-5	0.8556	0.7185	0.8448	0.4840	0.9298	0.8280	ns	ns	ns
ZT5-6	0.9404	0.9690	0.3321	0.0234	0.0760	0.0603	ns	ns	ns
ZT6-7	0.1678	0.3358	0.3128	<0.0001	0.0021	0.0216	ns	ns	ns
ZT7-8	0.0003	<0.0001	0.3743	0.9471	0.3120	0.0008	ns	ns	ns
ZT8-9	<0.0001	0.9511	<0.0001	0.4955	0.6085	0.4937	ns	ns	ns
ZT9-10	>0.9999	>0.9999	0.4020	<0.0001	0.1663	0.0455	ns	ns	ns
ZT10-11	0.0709	0.1667	0.3092	0.5090	0.0922	<0.0001	ns	ns	ns
ZT11-12	0.7331	>0.9999	0.8686	0.5517	0.1070	0.0468	ns	ns	ns
ZT12-13	0.5493	0.3579	0.0802	0.0011	0.0111	0.1171	ns	ns	ns
ZT13-14	0.6080	0.0061	0.3821	0.0269	0.9540	0.0093	ns	ns	ns
ZT14-15	0.3373	0.0355	0.3100	0.8050	0.3550	0.4580	ns	ns	ns
ZT15-16	0.3016	0.0022	0.0626	0.9605	0.9818	0.8952	ns	ns	ns
ZT16-17	0.0910	0.0106	0.0879	0.9966	0.3719	0.8301	ns	ns	ns
ZT17-18	0.0504	0.0047	0.0209	0.9989	0.7792	0.3368	ns	ns	ns
ZT18-19	0.0113	0.0060	0.0317	0.3184	0.4735	0.4365	ns	ns	ns
ZT19-20	0.0149	0.0032	0.0095	0.2424	>0.9999	0.1001	ns	ns	ns
ZT20-21	0.2533	0.0386	0.0144	0.2765	0.7969	0.1505	ns	ns	ns
ZT21-22	0.2597	0.3544	0.1959	0.9943	0.8940	0.9809	ns	ns	ns
ZT22-23	0.0024	0.0394	0.0985	0.3160	0.6696	0.6003	ns	ns	ns
ZT23-24	0.0170	0.0596	0.0096	0.1281	0.9342	0.0796	ns	ns	ns
ZT24-25	0.8929	0.9648	0.4735	0.0238	0.0136	0.1013	ns	ns	ns
ZT25-26	0.9853	0.9994	0.5683	0.0619	0.0246	0.0002	ns	ns	ns
ZT26-27	0.9853	0.2475	0.8228	0.9812	0.2330	0.0617	ns	ns	ns
ZT27-28	0.9771	0.7857	0.4495	0.2291	0.2123	0.7262	ns	ns	ns
ZT28-29	0.3856	0.9034	0.9803	0.0176	0.2097	0.0461	ns	ns	ns
ZT29-30	0.9851	0.6283	0.9927	<0.0001	0.1686	<0.0001	ns	ns	ns
ZT30-31	0.9122	0.3080	0.9723	<0.0001	<0.0001	<0.0001	ns	ns	ns

			Group		Time		Interaction	

	Data	DFn, DFd	F	p	F	p	F	p
Fig. 7J	Total sleep	2, 79	18.94	＜0.0001	178.8	＜0.0001	8.6940	＜0.0001
	Sleep episodes	2, 79	19.82	＜0.0001	16.39	＜0.0001	3.1660	＜0.0001
	P(wake)	2, 78	8.348	0.0005	137	＜0.0001	5.6740	＜0.0001

Dunnett's multiple comparisons

	MB043B/dTrpA1 vs MB043B/+			MB043B/dTrpA1 vs dTrpA1/+			Summary		
	Total sleep	Sleep episodes	P(wake)	Total sleep	Sleep episodes	P(wake)	Total sleep	Sleep episodes	P(wake)
ZT0-1	0.0012	0.0129	-	0.4400	0.7524	-	ns	ns	-
ZT1-2	0.0032	0.0029	0.0187	0.0697	0.1516	0.4706	ns	ns	ns
ZT2-3	0.0036	0.0019	0.0383	0.5696	0.8560	0.1921	ns	ns	ns
ZT3-4	0.0023	0.0041	0.0498	0.1846	0.1069	0.5094	ns	ns	ns
ZT4-5	0.8898	0.7974	0.0507	＜0.0001	0.9670	0.3653	ns	ns	ns
ZT5-6	0.3382	0.0357	0.5267	＜0.0001	0.2234	＜0.0001	ns	ns	ns
ZT6-7	0.0015	0.0618	0.0789	＜0.0001	0.0620	＜0.0001	**	ns	ns
ZT7-8	0.9979	0.3545	0.7467	0.0012	0.3976	＜0.0001	ns	ns	ns
ZT8-9	0.0076	0.1832	0.2104	＜0.0001	0.9648	0.0025	**	ns	ns
ZT9-10	0.8015	0.0893	0.8603	0.0517	0.3482	0.0090	ns	ns	ns
ZT10-11	0.0008	0.0024	0.1810	0.9587	0.8682	0.7601	ns	ns	ns
ZT11-12	0.0279	0.0354	0.1140	0.7040	0.4804	0.9664	ns	ns	ns
ZT12-13	0.5118	0.7019	0.5201	0.0638	0.4647	0.9425	ns	ns	ns
ZT13-14	0.0491	0.0836	0.0259	0.0020	0.9500	0.0062	*	ns	*
ZT14-15	0.0002	0.0038	0.0213	0.0002	0.0811	0.0093	***	ns	*
ZT15-16	0.0108	0.0147	0.0053	0.0003	0.0083	0.0026	*	*	**
ZT16-17	0.0155	0.0064	0.0830	0.0003	0.0011	0.0108	*	**	ns
ZT17-18	0.0028	＜0.0001	0.0059	0.0002	0.0002	0.0002	**	***	**
ZT18-19	0.0007	0.0014	0.0154	0.0002	0.0011	0.0014	***	**	*
ZT19-20	0.0019	0.0019	0.0011	0.0002	0.0016	0.0006	**	**	**
ZT20-21	0.0006	0.0020	0.0034	0.0032	0.0034	0.0063	**	**	**
ZT21-22	0.0005	0.0046	0.0023	0.0019	0.0095	0.0076	**	**	**
ZT22-23	0.0002	0.0008	0.0010	0.0005	0.0298	0.0077	***	*	**
ZT23-24	0.0007	0.0010	0.0019	0.0095	0.0182	0.0051	**	*	**
ZT24-25	0.0112	0.0016	0.0078	0.9656	0.9991	0.0116	ns	ns	*
ZT25-26	0.0065	0.0067	0.0952	0.6850	0.7371	0.5757	ns	ns	ns
ZT26-27	0.0076	0.3405	0.0501	0.0352	0.4913	0.4102	*	ns	ns
ZT27-28	0.0018	0.1981	0.0123	0.0628	0.9391	0.1087	ns	ns	ns
ZT28-29	0.0089	0.4440	0.1439	0.8539	0.7427	0.8064	ns	ns	ns
ZT29-30	0.0633	0.9574	0.0568	0.3596	0.4635	0.8512	ns	ns	ns
ZT30-31	0.4448	0.4818	0.7767	0.7838	0.4160	0.2171	ns	ns	ns

			Group		Time		Interaction	
	Data	DFn, DFd	F	p	F	p	F	p
Fig. 7K	Total sleep	2, 100	29.75	＜0.0001	214.3	＜0.0001	11.17	＜0.0001
	Sleep episodes	2, 100	13.18	＜0.0001	18.11	＜0.0001	5.3760	＜0.0001
	P(wake)	2, 101	10.23	＜0.0001	166.8	＜0.0001	9.9810	＜0.0001

Dunnett's multiple comparisons

	MB043B/shi^{ts1} vs MB043B/+			MB043B/shi^{ts1} vs shi^{ts1}/+			Summary		
	Total sleep	Sleep episodes	P(wake)	Total sleep	Sleep episodes	P(wake)	Total sleep	Sleep episodes	P(wake)
ZT0-1	0.7629	0.8750	0.8152	0.0292	0.3556	0.0004	ns	ns	ns
ZT1-2	0.2073	0.0745	0.9994	0.5546	0.3382	0.0505	ns	ns	ns
ZT2-3	0.3581	0.5553	0.0235	0.4590	0.5238	0.1837	ns	ns	ns
ZT3-4	0.0523	0.0154	0.2194	0.0142	0.0143	0.3313	ns	*	ns
ZT4-5	0.0198	0.1146	0.0303	<0.0001	0.4448	0.0404	*	ns	*
ZT5-6	<0.0001	0.1431	0.0077	<0.0001	0.8487	<0.0001	****	ns	**
ZT6-7	<0.0001	0.0808	<0.0001	<0.0001	0.7751	<0.0001	****	ns	****
ZT7-8	0.9826	0.6338	<0.0001	0.0002	>0.9999	<0.0001	ns	ns	****
ZT8-9	0.1016	0.8709	0.0020	0.0145	0.6063	0.0553	ns	ns	ns
ZT9-10	0.0085	>0.9999	0.6787	<0.0001	0.0324	<0.0001	**	ns	ns
ZT10-11	0.3917	0.5212	0.1024	0.0981	0.5159	<0.0001	ns	ns	ns
ZT11-12	0.1553	0.1340	0.4362	0.3605	0.1312	0.2042	ns	ns	ns
ZT12-13	0.4068	0.4866	0.7569	0.0564	0.0213	0.0500	ns	ns	ns
ZT13-14	0.0387	0.0043	0.9123	0.0605	0.7237	0.9910	ns	ns	ns
ZT14-15	<0.0001	0.0005	0.0007	0.0007	0.3045	0.0307	***	ns	*
ZT15-16	0.0001	<0.0001	<0.0001	<0.0001	0.2105	<0.0001	***	ns	****
ZT16-17	0.0008	<0.0001	<0.0001	0.0001	0.0009	<0.0001	***	***	****
ZT17-18	<0.0001	0.0003	0.0002	<0.0001	0.0161	<0.0001	****	*	***

ZT18-19	0.0011	<0.0001	0.0001	<0.0001	<0.0001	<0.0001	**	****	***
ZT19-20	0.0025	<0.0001	0.0017	<0.0001	0.0126	0.0001	**	*	**
ZT20-21	<0.0001	<0.0001	<0.0001	<0.0001	<0.0001	<0.0001	****	****	****
ZT21-22	0.0009	0.0002	<0.0001	<0.0001	<0.0001	<0.0001	***	***	****
ZT22-23	0.0019	<0.0001	0.0002	0.0066	0.0006	0.0003	**	***	***
ZT23-24	0.0002	<0.0001	0.0003	<0.0001	0.0089	0.0002	***	**	***
ZT24-25	0.9226	0.6206	0.1322	0.1509	0.1250	0.0025	ns	ns	ns
ZT25-26	0.4159	0.9544	0.8900	0.0498	0.0208	0.0055	ns	ns	ns
ZT26-27	0.6479	0.9408	0.6301	0.8007	0.5577	0.1041	ns	ns	ns
ZT27-28	0.1835	0.5260	0.2652	0.2042	0.4179	0.9925	ns	ns	ns
ZT28-29	0.0001	0.8840	0.0050	0.1684	0.9925	0.5242	ns	ns	ns
ZT29-30	0.0170	0.9084	0.0048	<0.0001	0.6428	0.0360	ns	ns	*
ZT30-31	0.0221	0.9918	0.0159	<0.0001	0.0201	<0.0001	ns	ns	ns

			Group		Time		Interaction	
	Data	DFn, DFd	F	p	F	p	F	p
Fig. 9A	Total sleep	3, 149	10.14	＜0.0001	129.9	＜0.0001	11.29	＜0.0001
	Sleep episodes	3, 149	26.05	＜0.0001	4.538	＜0.0001	3.516	0.0002
	P(wake)	3, 149	64.03	＜0.0001	72.10	＜0.0001	9.350	＜0.0001

Dunnett's multiple comparisons

	VT064246, MB299B/shi^{ts1} vs shi^{ts1}/+			MB299B/shi^{ts1} vs shi^{ts1}/+			VT064246/shi^{ts1} vs shi^{ts1}/+			Summary		
	Total sleep	Sleep episodes	P(wake)	Total sleep	Sleep episodes	P(wake)	Total sleep	Sleep episodes	P(wake)	Total sleep	Sleep episodes	P(wake)
ZT0-1	0.0012	0.0822	0.6939	0.4009	0.9999	0.7227	0.9699	0.9982	0.1135	ns	ns	ns
ZT1-2	0.6433	>0.9999	0.9528	0.9985	0.8127	0.9989	0.7121	0.9997	0.9997	ns	ns	ns
ZT2-3	0.0585	0.7961	0.0255	0.3372	0.8271	0.8464	0.0792	0.4275	0.1163	ns	ns	ns
ZT3-4	0.4668	0.1137	>0.9999	0.5251	0.9315	0.9486	0.0362	0.9550	0.4602	ns	ns	ns
ZT4-5	<0.0001	0.0433	<0.0001	<0.0001	0.9898	<0.0001	<0.0001	0.9008	<0.0001	****	ns	****
ZT5-6	0.0962	0.9763	<0.0001	0.9799	0.0140	0.6201	0.0334	>0.9999	0.0002	ns	ns	ns
ZT6-7	0.0001	0.4568	<0.0001	0.7972	0.4453	0.5538	0.0206	0.2832	0.0031	ns	ns	ns
ZT7-8	<0.0001	<0.0001	<0.0001	0.004	0.1856	0.2448	<0.0001	0.0015	0.0009	**	ns	ns
ZT8-9	<0.0001	0.3101	<0.0001	<0.0001	0.9990	<0.0001	<0.0001	0.5269	<0.0001	****	ns	****
ZT9-10	0.8679	0.0001	0.0824	0.1871	0.0037	0.9995	0.5547	0.0090	0.9404	ns	**	ns
ZT10-11	0.0002	<0.0001	<0.0001	0.0005	<0.0001	<0.0001	0.0079	0.0034	<0.0001	**	**	****
ZT11-12	0.0008	<0.0001	<0.0001	0.0026	0.0006	<0.0001	0.0400	0.0169	0.0022	*	*	**
ZT12-13	0.0004	0.9910	<0.0001	<0.0001	0.7508	<0.0001	<0.0001	0.4758	<0.0001	***	ns	****
ZT13-14	<0.0001	0.0002	<0.0001	<0.0001	0.0011	<0.0001	<0.0001	0.0021	<0.0001	****	**	****
ZT14-15	<0.0001	<0.0001	0.0139	<0.0001	<0.0001	0.0003	<0.0001	0.0046	<0.0001	****	**	*
ZT15-16	0.0002	<0.0001	0.0066	0.0002	0.0045	0.0011	<0.0001	0.0161	<0.0001	***	*	**
ZT16-17	<0.0001	<0.0001	0.0459	0.0001	0.0003	0.0153	<0.0001	0.0006	<0.0001	***	***	*
ZT17-18	0.0022	0.0003	0.0495	0.0018	0.0974	0.019	<0.0001	0.2057	<0.0001	**	ns	*
ZT18-19	0.0033	<0.0001	0.0241	0.0020	<0.0001	0.0847	<0.0001	0.0001	<0.0001	**	***	ns
ZT19-20	<0.0001	<0.0001	0.0859	0.0019	0.0003	0.4053	<0.0001	<0.0001	<0.0001	**	***	ns
ZT20-21	0.0016	<0.0001	0.1985	0.0844	0.0276	0.3787	<0.0001	<0.0001	0.0082	ns	*	ns
ZT21-22	0.0106	0.0003	0.6366	0.0897	0.0251	0.4458	<0.0001	<0.0001	0.0116	ns	*	ns
ZT22-23	0.4068	0.0099	0.8556	0.1296	0.1362	0.3387	0.0011	0.0081	0.0084	ns	ns	ns
ZT23-24	0.9757	0.0020	<0.0001	0.2522	0.0193	<0.0001	0.0263	0.0218	<0.0001	ns	*	****
ZT24-25	<0.0001	<0.0001	<0.0001	0.1589	0.0046	<0.0001	0.0047	0.0138	<0.0001	ns	*	****
ZT25-26	<0.0001	<0.0001	<0.0001	0.0778	0.0008	<0.0001	0.0495	0.8653	<0.0001	ns	ns	****
ZT26-27	<0.0001	0.0059	<0.0001	0.0807	0.0037	<0.0001	0.0115	0.4180	0.0002	ns	ns	***
ZT27-28	<0.0001	0.0230	<0.0001	0.0629	0.0066	0.535	0.0111	0.6860	0.6026	ns	ns	ns
ZT28-29	0.0003	0.0188	0.011	0.9546	0.0928	0.724	0.8995	0.7298	>0.9999	ns	ns	ns

			Group		Time		Interaction	
	Data	DFn, DFd	F	p	F	p	F	p
Fig. 9B	Total sleep	3, 116	1.191	0.3162	362.6	＜0.0001	5.321	＜0.0001
	Sleep episodes	3, 116	5.816	0.0010	24.25	＜0.0001	2.527	＜0.0001
	P(wake)	3, 116	13.07	＜0.0001	191.8	＜0.0001	5.645	＜0.0001

Dunnett's multiple comparisons

	VT064246, MB299B/shi^{ts1} vs shi^{ts1}/+			MB299B/shi^{ts1} vs shi^{ts1}/+			VT064246/shi^{ts1} vs shi^{ts1}/+			Summary		
	Total sleep	Sleep episodes	P(wake)	Total sleep	Sleep episodes	P(wake)	Total sleep	Sleep episodes	P(wake)	Total sleep	Sleep episodes	P(wake)
ZT0-1	0.0103	0.0017	<0.0001	0.0082	0.0043	<0.0001	0.0522	0.0556	0.0009	ns	ns	***
ZT1-2	0.0118	0.0007	<0.0001	0.0037	0.0006	<0.0001	0.3924	0.3516	0.1495	ns	ns	ns
ZT2-3	0.2496	0.9481	0.4385	0.9134	0.9421	0.7102	0.0055	0.0327	<0.0001	ns	ns	ns

ZT3-4	0.1313	0.9997	0.0053	0.9615	0.6141	0.3147	0.0005	0.8393	<0.0001	ns	ns	ns
ZT4-5	<0.0001	0.7066	<0.0001	<0.0001	0.5016	<0.0001	<0.0001	0.8397	<0.0001	****	ns	****
ZT5-6	0.5137	0.5190	0.2726	0.0048	0.0576	0.9944	0.5146	>0.9999	0.0112	ns	ns	ns
ZT6-7	0.2615	0.2026	0.7645	0.1905	0.0009	0.6368	0.6394	0.1354	0.5593	ns	ns	ns
ZT7-8	0.0123	0.0077	0.1112	0.1023	0.0581	0.1758	0.4824	0.9989	0.5433	ns	ns	ns
ZT8-9	0.7349	0.0093	0.8624	>0.9999	0.6661	0.963	0.1297	0.0084	0.1596	ns	ns	ns
ZT9-10	0.3777	0.1718	0.0706	0.4230	0.1025	0.1385	0.0136	0.0331	0.0006	ns	ns	ns
ZT10-11	0.1639	0.0055	0.2396	0.4143	0.0769	0.3056	0.1275	0.0316	0.0731	ns	ns	ns
ZT11-12	0.0035	0.0013	<0.0001	0.0051	0.0085	<0.0001	0.1701	0.1295	0.0001	ns	ns	***
ZT12-13	0.9002	0.9165	0.8841	0.2025	0.1386	0.0027	0.9968	0.1032	0.6822	ns	ns	ns
ZT13-14	0.3590	0.0660	0.4135	0.0027	0.0305	0.0062	0.0162	0.0035	0.1486	ns	ns	ns
ZT14-15	0.3260	0.1938	0.7823	0.0125	0.0062	0.437	0.2132	0.1447	0.7811	ns	ns	ns
ZT15-16	0.0681	0.1197	0.6239	0.0057	0.0677	0.5947	0.0302	0.0703	0.8581	ns	ns	ns
ZT16-17	0.3349	0.8176	0.9227	0.1069	0.0465	0.763	0.4191	0.1614	0.9277	ns	ns	ns
ZT17-18	0.7686	0.8747	0.9730	0.3623	0.0955	0.8591	0.7458	0.4303	0.9661	ns	ns	ns
ZT18-19	0.2418	0.3409	0.8494	0.0706	0.0501	0.9412	0.1038	0.3003	0.6700	ns	ns	ns
ZT19-20	0.1315	0.9081	0.8608	0.1567	0.6220	0.9717	0.1203	0.9979	0.9467	ns	ns	ns
ZT20-21	0.2492	0.7762	0.9562	0.9919	0.3888	>0.9999	0.6921	0.9825	>0.9999	ns	ns	ns
ZT21-22	0.4072	0.9972	0.7979	0.8373	0.9045	>0.9999	0.9271	0.7844	0.9960	ns	ns	ns
ZT22-23	0.9986	0.5470	0.9999	0.8669	0.9988	0.9958	0.8884	0.9867	0.9966	ns	ns	ns
ZT23-24	<0.0001	0.0252	0.3332	0.0073	0.9617	0.6343	<0.0001	0.9089	0.4822	**	ns	ns
ZT24-25	0.0004	<0.0001	<0.0001	0.0002	0.0006	<0.0001	0.0147	0.0716	0.0035	*	ns	**
ZT25-26	0.0239	0.0014	<0.0001	0.0025	<0.0001	<0.0001	0.6768	0.0498	0.1390	ns	*	ns
ZT26-27	0.9932	0.1633	0.7489	0.2377	0.0081	0.2974	0.3914	0.7054	0.0420	ns	ns	ns
ZT27-28	0.0007	0.9996	0.0018	0.3792	0.3802	0.3490	0.0005	0.5244	<0.0001	ns	ns	ns
ZT28-29	0.0041	0.8965	0.0254	0.0534	0.4718	0.2205	<0.0001	0.9933	<0.0001	ns	ns	ns

			Group		Time		Interaction	
	Data	DFn, DFd	F	p	F	p	F	p
Fig. 9C	Total sleep	2, 121	5.4550	0.0054	163.3	＜0.0001	4.773	＜0.0001
	Sleep episodes	2, 121	46.12	＜0.0001	53.63	＜0.0001	2.890	＜0.0001
	P(wake)	2, 121	28.78	＜0.0001	111.8	＜0.0001	4.606	＜0.0001

THIP

Dunnett's multiple comparisons

	MB299B/dTrpA1 vs MB299B/+			MB299B/dTrpA1 vs dTrpA1/+			Summary		
	Total sleep	Sleep episodes	P(wake)	Total sleep	Sleep episodes	P(wake)	Total sleep	Sleep episodes	P(wake)
ZT0-1	>0.9999	>0.9999	>0.9999	>0.9999	>0.9999	>0.9999	ns	ns	ns
ZT1-2	<0.0001	0.0007	<0.0001	0.0015	0.0015	<0.0001	**	**	****
ZT2-3	0.0025	0.0020	0.0002	0.9165	0.0001	0.0018	ns	**	**
ZT3-4	0.4794	0.0039	0.4226	0.2734	<0.0001	0.0002	ns	**	ns
ZT4-5	0.1300	0.8895	0.1198	0.9998	0.0203	0.3675	ns	ns	ns
ZT5-6	0.0066	0.0002	0.0003	0.9625	<0.0001	0.8478	ns	***	ns
ZT6-7	0.1271	<0.0001	0.1878	0.9319	0.0007	0.5117	ns	***	ns
ZT7-8	0.0053	0.0196	0.0102	0.1474	0.0003	0.1118	ns	*	ns
ZT8-9	0.8029	0.2057	0.6601	0.0047	<0.0001	0.0051	ns	ns	ns
ZT9-10	0.1210	<0.0001	0.1673	0.7838	<0.0001	0.0794	ns	****	ns
ZT10-11	0.2984	0.4497	0.8538	0.1240	0.0025	0.8052	ns	ns	ns
ZT11-12	0.1363	0.9995	0.986	0.0353	0.7733	0.9802	ns	ns	ns
ZT12-13	0.8437	0.0020	0.9424	0.0054	0.1270	0.0187	ns	ns	ns
ZT13-14	0.9803	0.9990	0.9752	0.2276	0.6382	0.8253	ns	ns	ns
ZT14-15	0.2842	0.6901	0.928	0.5218	0.9982	0.9938	ns	ns	ns
ZT15-16	0.1458	0.5454	0.8994	0.0301	0.1401	0.6789	ns	ns	ns
ZT16-17	0.9067	0.9110	0.9992	0.7096	0.5752	0.8705	ns	ns	ns
ZT17-18	0.8000	0.5803	0.9404	0.9212	>0.9999	0.9924	ns	ns	ns
ZT18-19	0.9836	0.9874	0.9978	0.5325	0.9044	0.9406	ns	ns	ns
ZT19-20	0.7899	0.9843	0.9936	0.6374	0.9273	0.9577	ns	ns	ns
ZT20-21	0.0596	0.5384	0.9297	0.1306	0.4757	0.6704	ns	ns	ns
ZT21-22	0.3002	0.8908	0.8979	0.2139	0.7441	0.8780	ns	ns	ns
ZT22-23	0.9757	0.9758	0.9983	0.8121	0.9554	0.9193	ns	ns	ns
ZT23-24	0.1441	0.6972	0.8504	0.1902	0.9993	0.5056	ns	ns	ns
ZT24-25	<0.0001	0.9833	<0.0001	0.0002	0.8403	<0.0001	***	ns	****
ZT25-26	0.1190	0.7561	0.0314	0.0024	0.9860	<0.0001	ns	ns	*
ZT26-27	0.9528	0.5715	0.5897	0.0185	0.3839	<0.0001	ns	ns	ns
ZT27-28	0.1630	0.0010	0.0131	0.0295	0.0009	<0.0001	ns	***	*
ZT28-29	0.2208	0.7238	0.2136	0.5167	0.1863	0.2903	ns	ns	ns
ZT29-30	0.9525	0.2923	0.8716	0.0403	0.0289	0.0206	ns	ns	ns

ZT30-31	0.7100	0.8909	0.8372	0.0642	0.0258	0.0041	ns	ns	ns

			Group		Time		Interaction	
	Data	DFn, DFd	F	p	F	p	F	p
	Total sleep	2, 120	11.28	＜0.0001	187.6	＜0.0001	4.921	＜0.0001
Fig. 9D	Sleep episodes	2, 120	136.6	＜0.0001	24.47	＜0.0001	2.663	＜0.0001
	P(wake)	2, 120	68.03	＜0.0001	115.5	＜0.0001	3.822	＜0.0001

No THIP	Dunnett's multiple comparisons								
	MB299B/dTrpA1 vs MB299B/+			MB299B/dTrpA1 vs dTrpA1/+			Summary		
	Total sleep	Sleep episodes	P(wake)	Total sleep	Sleep episodes	P(wake)	Total sleep	Sleep episodes	P(wake)
ZT0-1	0.0000	>0.9999	>0.9999	0.0000	>0.9999	>0.9999	ns	ns	ns
ZT1-2	<0.0001	<0.0001	<0.0001	0.3155	0.0098	0.1434	ns	**	ns
ZT2-3	0.3188	0.0621	0.1293	0.9686	0.0345	0.2843	ns	ns	ns
ZT3-4	0.4801	0.1031	0.499	0.5186	0.1391	>0.9999	ns	ns	ns
ZT4-5	0.0058	0.0044	0.0177	0.3284	0.0375	0.9048	ns	*	ns
ZT5-6	0.0052	0.0004	0.0120	0.2461	0.0003	0.3825	ns	***	ns
ZT6-7	0.0349	0.1035	0.0179	0.5165	0.8308	0.3524	ns	ns	ns
ZT7-8	0.3903	0.5888	0.5023	<0.0001	0.1743	<0.0001	ns	ns	ns
ZT8-9	0.0003	0.0939	0.0016	<0.0001	<0.0001	<0.0001	***	ns	**
ZT9-10	0.4517	0.6421	0.6328	0.1320	0.1189	0.1644	ns	ns	ns
ZT10-11	0.5145	0.2046	0.9002	0.9614	0.2560	0.7398	ns	ns	ns
ZT11-12	0.8946	0.8603	0.7208	0.7045	0.7044	0.5085	ns	ns	ns
ZT12-13	0.3118	0.3101	0.0777	0.2076	0.8004	0.0322	ns	ns	ns
ZT13-14	0.0044	0.0002	0.0090	0.0002	0.0005	0.0019	**	***	**
ZT14-15	0.0070	0.0154	0.0187	0.1159	0.4901	0.0699	ns	ns	ns
ZT15-16	0.0894	0.1660	0.2314	0.0439	0.6076	0.1956	ns	ns	ns
ZT16-17	0.2326	0.4380	0.5854	0.1302	0.8774	0.5638	ns	ns	ns
ZT17-18	0.2170	0.7168	0.6225	0.2571	0.8930	0.6692	ns	ns	ns
ZT18-19	0.3937	>0.9999	0.8572	0.8981	0.9924	0.9642	ns	ns	ns
ZT19-20	0.2576	0.6006	0.6305	0.6617	0.9979	0.8228	ns	ns	ns
ZT20-21	0.0276	0.8190	0.2093	0.3351	0.7864	0.5658	ns	ns	ns
ZT21-22	0.3564	0.9404	0.7808	0.9997	0.8333	0.9989	ns	ns	ns
ZT22-23	0.9545	0.7168	0.9888	0.3941	0.4378	0.7727	ns	ns	ns
ZT23-24	0.3011	0.4537	0.8086	0.6479	0.5679	0.9384	ns	ns	ns
ZT24-25	0.6425	0.7526	0.0360	0.1782	0.5018	0.0042	ns	ns	*
ZT25-26	0.0145	0.9453	0.0014	0.0987	0.0664	0.0074	ns	ns	**
ZT26-27	0.0069	0.6050	<0.0001	0.4597	0.0752	0.0924	ns	ns	ns
ZT27-28	0.0110	0.1354	0.0008	0.1130	0.0263	0.0120	ns	ns	*
ZT28-29	0.0002	0.8988	<0.0001	0.0033	0.0454	<0.0001	**	ns	****
ZT29-30	<0.0001	0.5663	<0.0001	0.0004	0.0084	<0.0001	***	ns	****
ZT30-31	<0.0001	0.0666	<0.0001	<0.0001	0.0007	<0.0001	****	ns	****

			Group		Time		Interaction	
	Data	DFn, DFd	F	p	F	p	F	p
	Total sleep	2, 86	28.77	＜0.0001	75.90	＜0.0001	6.104	＜0.0001
Fig. 11A	Sleep episodes	2, 86	13.09	＜0.0001	5.620	＜0.0001	3.299	＜0.0001
	P(wake)	2, 86	33.61	＜0.0001	61.77	＜0.0001	5.769	＜0.0001

	Dunnett's multiple comparisons								
	MB310C/dTrpA1 vs MB310C/+			MB310C/dTrpA1 vs dTrpA1/+			Summary		
	Total sleep	Sleep episodes	P(wake)	Total sleep	Sleep episodes	P(wake)	Total sleep	Sleep episodes	P(wake)
ZT0-1	0.9209	0.8212	0.9727	>0.9999	0.9125	0.3328	ns	ns	ns
ZT1-2	0.9739	0.9054	0.8915	0.7835	0.3355	0.6966	ns	ns	ns
ZT2-3	0.9282	0.8102	0.9535	0.7731	0.2023	0.5547	ns	ns	ns
ZT3-4	0.9991	0.7525	0.9885	0.9569	0.5942	0.9750	ns	ns	ns
ZT4-5	0.9772	0.2479	0.8653	0.9943	0.7517	0.9871	ns	ns	ns
ZT5-6	0.2201	0.1629	0.0612	0.0243	0.0002	0.0073	ns	ns	ns
ZT6-7	0.8267	0.1489	0.9766	0.7558	0.951	0.843	ns	ns	ns
ZT7-8	0.9615	0.8731	0.9956	0.0278	0.0037	0.0045	ns	ns	ns
ZT8-9	0.9966	0.7818	0.2757	0.0719	<0.0001	0.0007	ns	ns	ns
ZT9-10	0.9700	0.8157	0.7109	0.0825	0.0001	0.0036	ns	ns	ns
ZT10-11	0.9608	0.7226	0.5655	0.6760	0.2394	0.1232	ns	ns	ns
ZT11-12	>0.9999	0.9985	0.9268	0.6941	0.1747	0.1854	ns	ns	ns
ZT12-13	0.4999	0.4009	0.0879	0.7045	0.5522	0.7301	ns	ns	ns
ZT13-14	0.0144	0.9545	0.0398	0.2463	0.2266	0.2253	ns	ns	ns
ZT14-15	0.2824	0.9829	0.3489	0.1192	0.2082	0.1915	ns	ns	ns

ZT15-16	0.0708	0.4962	0.2753	0.1845	0.3273	0.4015	ns	ns	ns
ZT16-17	0.0443	0.3957	0.1712	0.3006	0.1052	0.5685	ns	ns	ns
ZT17-18	0.2605	0.2765	0.5275	0.1293	0.3194	0.3575	ns	ns	ns
ZT18-19	0.0608	0.4502	0.4503	0.1545	0.0556	0.2011	ns	ns	ns
ZT19-20	0.1506	0.5139	0.7401	0.0511	0.0082	0.1124	ns	ns	ns
ZT20-21	0.2076	0.4447	0.3850	0.1263	0.0309	0.4961	ns	ns	ns
ZT21-22	0.4032	0.5139	0.4121	0.1451	0.2461	0.5181	ns	ns	ns
ZT22-23	0.2872	0.8730	0.4268	0.9456	0.0178	0.8179	ns	ns	ns
ZT23-24	0.4082	0.8873	0.6733	0.5687	0.0393	0.8829	ns	ns	ns
ZT24-25	0.5076	0.8320	0.9224	0.9954	0.7415	0.7698	ns	ns	ns
ZT25-26	0.324	0.1284	0.4005	0.4397	0.5522	0.4096	ns	ns	ns
ZT26-27	0.1700	0.3164	0.3728	0.4213	0.9889	0.5227	ns	ns	ns
ZT27-28	0.1106	0.1572	0.1624	0.6258	0.5215	0.6858	ns	ns	ns
ZT28-29	0.0240	0.3211	0.0181	0.4736	0.5011	0.2201	ns	ns	ns
ZT29-30	0.0164	0.2984	0.0353	0.4617	0.9510	0.2879	ns	ns	ns
ZT30-31	0.0032	0.9220	0.0962	0.4974	0.7618	0.1488	ns	ns	ns

			Group		Time		Interaction	
	Data	DFn, DFd	F	p	F	p	F	p
Fig. 11B	Total sleep	2, 120	30.75	＜0.0001	75.57	＜0.0001	8.833	＜0.0001
	Sleep episodes	2, 120	34.41	＜0.0001	8.902	＜0.0001	2.992	＜0.0001
	P(wake)	2, 120	47.69	＜0.0001	73.51	＜0.0001	9.187	＜0.0001

Dunnett's multiple comparisons

	MB310C/shi^{ts1} vs MB310C/+			MB310C/shi^{ts1} vs shi^{ts1}/+			Summary		
	Total sleep	Sleep episodes	P(wake)	Total sleep	Sleep episodes	P(wake)	Total sleep	Sleep episodes	P(wake)
ZT0-1	0.8249	0.9125	0.6284	0.6978	0.8573	0.1424	ns	ns	ns
ZT1-2	0.9926	0.9731	0.9487	0.8521	0.7287	0.9031	ns	ns	ns
ZT2-3	0.9967	0.1758	0.8049	0.9925	0.7195	0.3323	ns	*	ns
ZT3-4	0.6008	0.1189	0.2749	0.7758	0.2411	0.963	ns	ns	ns
ZT4-5	0.7972	0.0041	0.4624	0.1953	0.2762	0.1483	ns	ns	ns
ZT5-6	0.5537	0.4074	0.253	0.6152	0.0177	0.6434	ns	ns	ns
ZT6-7	0.0018	0.8219	0.0022	0.7723	0.2663	0.6300	ns	ns	ns
ZT7-8	0.7803	0.8340	0.9477	0.7120	0.9266	0.8216	ns	ns	ns
ZT8-9	0.2172	0.0012	0.1801	<0.0001	0.0374	<0.0001	ns	*	ns
ZT9-10	0.0408	0.0011	<0.0001	<0.0001	0.0309	<0.0001	*	*	****
ZT10-11	0.3039	0.0038	0.0014	0.0017	0.0109	<0.0001	ns	*	**
ZT11-12	0.6036	0.0226	0.0022	0.7658	0.6119	0.7890	ns	ns	ns
ZT12-13	<0.0001	0.0005	<0.0001	0.0001	0.2813	<0.0001	***	ns	****
ZT13-14	<0.0001	0.7580	<0.0001	<0.0001	0.0634	<0.0001	****	ns	****
ZT14-15	<0.0001	0.0089	<0.0001	<0.0001	0.1252	<0.0001	****	ns	****
ZT15-16	<0.0001	0.0214	<0.0001	<0.0001	0.0002	<0.0001	****	*	****
ZT16-17	<0.0001	0.0136	<0.0001	<0.0001	0.0093	<0.0001	****	*	****
ZT17-18	<0.0001	0.1862	<0.0001	<0.0001	0.1265	<0.0001	****	ns	****
ZT18-19	<0.0001	0.0023	<0.0001	<0.0001	0.0044	<0.0001	****	**	****
ZT19-20	<0.0001	0.0015	<0.0001	<0.0001	<0.0001	<0.0001	****	**	****
ZT20-21	<0.0001	0.0025	<0.0001	<0.0001	<0.0001	<0.0001	****	**	****
ZT21-22	0.0025	0.0215	0.008	0.0009	<0.0001	0.0124	**	*	*
ZT22-23	0.1899	0.0152	0.2984	0.1442	0.0027	0.3396	ns	*	ns
ZT23-24	0.0331	0.0110	0.0879	0.0361	0.0026	0.2144	*	*	ns
ZT24-25	0.9927	0.7842	0.5996	0.8293	0.5952	0.2000	ns	ns	ns
ZT25-26	0.9907	0.5504	0.8577	0.8694	0.9369	0.6275	ns	ns	ns
ZT26-27	0.9713	0.1650	0.7899	0.8366	0.7714	0.6709	ns	ns	ns
ZT27-28	0.9775	0.6379	0.7312	0.6895	0.8043	0.6911	ns	ns	ns
ZT28-29	0.9393	0.8604	0.9958	0.7253	0.4614	0.6374	ns	ns	ns

			Group		Time		Interaction	
	Data	DFn, DFd	F	p	F	p	F	p
Fig. 11C	Total sleep	2, 93	62.96	＜0.0001	227.6	＜0.0001	4.132	＜0.0001
	Sleep episodes	2, 93	32.60	＜0.0001	15.14	＜0.0001	1.679	＜0.0001
	P(wake)	2, 93	79.54	＜0.0001	187.9	＜0.0001	4.915	＜0.0001

Dunnett's multiple comparisons

	MB310C/dTrpA1 vs MB310C/+			MB310C/dTrpA1 vs dTrpA1/+			Summary		
	Total sleep	Sleep episodes	P(wake)	Total sleep	Sleep episodes	P(wake)	Total sleep	Sleep episodes	P(wake)
ZT0-1	0.3981	0.7024	0.5048	0.0008	0.0020	<0.0001	ns	ns	ns
ZT1-2	0.4935	0.9603	0.0265	<0.0001	0.0030	<0.0001	ns	ns	*

ZT2-3	0.0195	0.2692	0.0164	<0.0001	0.0473	<0.0001	*	ns	*
ZT3-4	0.1281	0.2693	0.0536	0.9248	0.0064	0.9872	ns	ns	ns
ZT4-5	0.0006	0.9603	0.0118	0.7230	0.7803	0.9252	ns	ns	ns
ZT5-6	0.8092	0.3274	0.7552	0.8155	0.7803	0.9717	ns	ns	ns
ZT6-7	0.8855	0.0256	0.7206	0.5477	0.1750	0.5827	ns	ns	ns
ZT7-8	0.0159	0.0631	<0.0001	0.7433	0.9133	0.9996	ns	ns	ns
ZT8-9	0.0212	0.0831	<0.0001	0.6106	0.8520	0.9039	ns	ns	ns
ZT9-10	0.1337	0.1384	0.0005	0.5963	0.3926	0.1859	ns	ns	ns
ZT10-11	0.4672	0.1079	0.0279	0.7160	0.9603	0.4684	ns	ns	ns
ZT11-12	0.6810	0.6214	0.4731	0.9636	>0.9999	0.8818	ns	ns	ns
ZT12-13	0.2161	0.9603	0.1588	0.6810	0.0631	0.9781	ns	ns	ns
ZT13-14	0.0007	0.6214	0.0206	0.1254	>0.9999	0.2154	ns	ns	ns
ZT14-15	0.0141	0.2692	0.1063	0.0009	0.0064	0.0402	*	ns	ns
ZT15-16	0.0305	0.4644	0.2660	0.0133	0.4644	0.2675	*	ns	ns
ZT16-17	0.3516	0.9603	0.7082	0.5339	0.9133	0.8570	ns	ns	ns
ZT17-18	0.5068	0.9603	0.7770	0.9727	0.9899	>0.9999	ns	ns	ns
ZT18-19	0.9727	0.4644	0.9797	0.9923	0.7024	0.9855	ns	ns	ns
ZT19-20	0.7365	0.8520	0.9385	0.9667	0.9603	0.9858	ns	ns	ns
ZT20-21	0.4351	0.1750	0.7562	0.6317	0.6214	0.7603	ns	ns	ns
ZT21-22	0.9938	0.0631	0.9469	0.9568	0.8520	0.9959	ns	ns	ns
ZT22-23	0.7020	0.5413	0.8929	0.4164	>0.9999	0.8509	ns	ns	ns
ZT23-24	0.8092	0.7024	0.9290	0.9495	0.7803	0.9978	ns	ns	ns
ZT24-25	0.0055	0.0043	<0.0001	<0.0001	<0.0001	<0.0001	**	**	****
ZT25-26	0.0005	0.7025	0.0004	<0.0001	0.0001	<0.0001	***	ns	***
ZT26-27	0.6388	0.3925	0.4132	<0.0001	0.2184	<0.0001	ns	ns	ns
ZT27-28	0.0039	0.6214	0.0789	0.0060	0.0256	0.009	**	ns	ns
ZT28-29	0.0035	0.7022	0.0077	0.9418	0.7025	0.9923	ns	ns	ns
ZT29-30	0.0003	0.2186	0.0061	0.0297	0.6214	0.1137	*	ns	ns
ZT30-31	<0.0001	>0.9999	0.0011	0.0028	0.9899	0.0090	**	ns	**

	Data	DFn, DFd	Group F	Group p	Time F	Time p	Interaction F	Interaction p
Fig. 11D	Total sleep	2, 124	394.4	＜0.0001	249.6	＜0.0001	7.587	＜0.0001
	Sleep episodes	2, 124	54.30	＜0.0001	21.67	＜0.0001	3.888	＜0.0001
	P(wake)	2, 124	315.5	＜0.0001	246.7	＜0.0001	9.371	＜0.0001

Dunnett's multiple comparisons

	MB310C/shi^{ts1} vs MB310C/+			MB310C/shi^{ts1} vs shi^{ts1}/+			Summary		
	Total sleep	Sleep episodes	P(wake)	Total sleep	Sleep episodes	P(wake)	Total sleep	Sleep episodes	P(wake)
ZT0-1	<0.0001	0.1314	<0.0001	0.4594	0.3620	<0.0001	ns	ns	****
ZT1-2	<0.0001	0.9305	<0.0001	0.6375	0.3515	0.0138	ns	ns	*
ZT2-3	<0.0001	0.9919	<0.0001	0.1509	0.3771	0.6089	ns	ns	ns
ZT3-4	<0.0001	0.4696	<0.0001	<0.0001	0.8699	<0.0001	****	ns	****
ZT4-5	<0.0001	0.0003	<0.0001	<0.0001	0.0879	0.0003	****	ns	***
ZT5-6	0.0919	<0.0001	0.3379	0.0002	0.0170	0.2468	ns	*	ns
ZT6-7	0.1917	0.9919	0.4836	0.1244	0.8319	0.3470	ns	ns	ns
ZT7-8	0.0365	0.1050	0.2912	0.4234	0.0889	0.7433	ns	ns	ns
ZT8-9	<0.0001	0.0502	<0.0001	<0.0001	0.0018	<0.0001	****	ns	****
ZT9-10	<0.0001	0.0119	<0.0001	<0.0001	0.0039	<0.0001	****	*	****
ZT10-11	<0.0001	0.2420	<0.0001	0.0050	0.3356	<0.0001	**	ns	****
ZT11-12	<0.0001	0.1995	<0.0001	0.8301	0.6909	0.4675	ns	ns	ns
ZT12-13	<0.0001	0.6102	<0.0001	<0.0001	0.2493	<0.0001	****	ns	****
ZT13-14	<0.0001	<0.0001	0.0002	<0.0001	<0.0001	0.0001	****	****	***
ZT14-15	<0.0001	<0.0001	0.0112	<0.0001	<0.0001	0.002	****	****	*
ZT15-16	0.0006	0.0031	0.0577	0.0007	0.0002	0.0524	***	**	ns
ZT16-17	<0.0001	0.0062	0.0217	<0.0001	0.0053	0.0265	****	**	*
ZT17-18	0.0198	0.0086	0.2010	0.0163	0.0039	0.1824	*	**	ns
ZT18-19	0.0062	0.0007	0.0936	0.0037	0.0011	0.0909	**	**	ns
ZT19-20	0.4957	0.2903	0.8552	0.3081	0.0185	0.7265	ns	ns	ns
ZT20-21	0.3988	0.1314	0.7413	0.3662	0.0469	0.6987	ns	ns	ns
ZT21-22	0.9763	0.8202	0.9925	0.9581	0.9219	0.9977	ns	ns	ns
ZT22-23	0.6599	0.3446	0.8807	0.7810	0.9491	0.9537	ns	ns	ns
ZT23-24	0.9980	0.9683	0.9986	>0.9999	>0.9999	0.9980	ns	ns	ns
ZT24-25	<0.0001	0.4046	<0.0001	0.8635	0.7294	0.0451	ns	ns	*
ZT25-26	<0.0001	0.0022	<0.0001	0.0491	0.4102	0.3330	*	ns	ns
ZT26-27	<0.0001	0.9683	<0.0001	<0.0001	0.0576	0.0013	****	ns	**
ZT27-28	<0.0001	0.0829	<0.0001	<0.0001	0.8104	<0.0001	****	ns	****

ZT28-29	<0.0001	0.0022	<0.0001	<0.0001	0.4197	<0.0001	****	ns	****

	Data	DFn, DFd	Group F	Group p	Time F	Time p	Interaction F	Interaction p
Figure S1A	Total sleep	2, 122	3.663	0.0285	542.2	＜0.0001	4.285	＜0.0001
	Sleep episodes	2, 122	3.395	0.0367	25.91	＜0.0001	2.238	＜0.0001
	P(wake)	2, 109	8.903	0.0003	270.2	＜0.0001	3.895	＜0.0001

	c316/dTrpA1 vs c316/+			c316/dTrpA1 vs dTrpA1/+			Summary		
	Total sleep	Episodes	P(wake)	Total sleep	Episodes	P(wake)	Total sleep	Episodes	P(wake)
ZT0-1	-	-	0.2729	-	-	0.9731	-	-	ns
ZT1-2	0.0416	0.0449	0.0251	0.9944	0.7676	0.8389	ns	ns	ns
ZT2-3	0.0238	0.0079	0.0661	0.6571	0.6346	0.9494	ns	ns	ns
ZT3-4	0.9595	0.8212	0.7928	0.9883	0.9244	0.7709	ns	ns	ns
ZT4-5	0.6517	0.2818	0.6146	0.7396	0.9968	0.1197	ns	ns	ns
ZT5-6	0.4275	0.9773	0.9887	0.0490	0.7132	0.0131	ns	ns	ns
ZT6-7	0.2991	0.4860	0.7434	0.0009	0.7980	0.9988	ns	ns	ns
ZT7-8	0.0009	0.6351	0.2941	＜0.0001	0.1021	0.0313	***	ns	ns
ZT8-9	0.3708	0.8332	0.9429	0.9635	0.0576	0.1104	ns	ns	ns
ZT9-10	0.9755	0.0910	0.3739	0.0975	0.3645	0.0268	ns	ns	ns
ZT10-11	0.3291	0.0958	0.0237	0.1601	0.4534	0.1541	ns	ns	ns
ZT11-12	0.7080	0.3258	0.7644	0.0099	0.0085	0.0033	ns	ns	ns
ZT12-13	0.6876	0.7817	0.0611	0.0070	0.3628	<0.0001	ns	ns	ns
ZT13-14	0.0471	0.2383	0.264	＜0.0001	0.0007	0.0115	*	ns	ns
ZT14-15	0.0525	0.1282	0.0689	0.0241	0.0020	0.0302	ns	ns	ns
ZT15-16	0.1835	0.9479	0.0906	0.1267	0.3157	0.1077	ns	ns	ns
ZT16-17	0.0482	0.2292	0.2923	0.6146	0.0307	0.3538	ns	ns	ns
ZT17-18	0.6288	0.8345	0.8326	0.7372	0.7873	0.5124	ns	ns	ns
ZT18-19	0.7262	0.9503	0.2319	0.8776	0.9609	0.7318	ns	ns	ns
ZT19-20	0.0933	0.1098	0.5267	0.8844	0.9567	0.5553	ns	ns	ns
ZT20-21	0.4664	0.7729	0.2610	0.9652	＞0.9999	0.3811	ns	ns	ns
ZT21-22	0.0684	0.2100	0.0467	0.3909	0.0835	0.4988	ns	ns	ns
ZT22-23	0.1829	0.2046	0.2136	0.1383	0.1650	0.0132	ns	ns	ns
ZT23-24	0.2747	0.5565	0.4119	0.0209	0.3919	0.0067	ns	ns	ns
ZT24-25	0.1774	0.0525	0.0154	0.9825	0.8761	0.4027	ns	ns	ns
ZT25-26	0.2638	0.2641	0.0279	0.5260	0.3123	0.5431	ns	ns	ns
ZT26-27	0.9986	0.9976	0.2889	0.1335	0.1367	0.7433	ns	ns	ns
ZT27-28	0.9416	0.9997	0.3050	0.8498	0.7472	0.6701	ns	ns	ns
ZT28-29	0.6998	0.4309	0.0702	0.5270	0.5210	0.5182	ns	ns	ns
ZT29-30	0.2991	0.2914	0.0647	0.3147	0.8440	0.1195	ns	ns	ns
ZT30-31	0.3330	0.7330	0.1081	0.0336	0.8739	0.3067	ns	ns	ns

	Data	DFn, DFd	Group F	Group p	Time F	Time p	Interaction F	Interaction p
Figure S2A	Total sleep	2, 83	12.16	＜0.0001	180.6	＜0.0001	4.2060	＜0.0001
	Sleep episodes	2, 83	10.90	＜0.0001	15.79	＜0.0001	2.8380	＜0.0001
	P(wake)	2, 84	20.33	＜0.0001	158.3	＜0.0001	4.5350	＜0.0001

Dunnett's multiple comparisons

	MB299B/dTrpA1 vs MB299B/+			MB299B/dTrpA1 vs dTrpA1/+			Summary		
	Total sleep	Sleep episodes	P(wake)	Total sleep	Sleep episodes	P(wake)	Total sleep	Sleep episodes	P(wake)
ZT0-1	0.9271	0.8150	0.9569	0.0025	0.0032	0.2554	ns	ns	ns
ZT1-2	0.0380	0.0005	0.5383	0.3502	0.3906	0.0436	ns	ns	ns
ZT2-3	0.0345	0.3404	0.0151	0.6377	0.4221	0.1726	ns	ns	ns
ZT3-4	＜0.0001	0.0091	0.0008	0.4268	0.2148	0.5099	ns	ns	ns
ZT4-5	0.0026	0.1054	0.0008	0.2269	0.4578	0.5939	ns	ns	ns
ZT5-6	＜0.0001	0.8922	＜0.0001	0.0062	0.6089	0.0024	**	ns	**
ZT6-7	＜0.0001	0.5051	＜0.0001	0.0188	0.5812	0.2621	*	ns	ns
ZT7-8	0.6767	0.9945	0.0488	0.0081	0.7022	0.0039	ns	ns	*
ZT8-9	0.0005	0.0533	0.0046	0.1411	0.9933	0.1829	ns	ns	ns
ZT9-10	0.3437	0.5029	0.0261	0.2293	0.0146	0.1745	ns	ns	ns
ZT10-11	0.5884	0.3553	0.3099	0.0125	0.1773	0.0407	ns	ns	ns
ZT11-12	0.0227	0.0746	0.0095	0.1394	0.2117	0.0179	ns	ns	ns
ZT12-13	0.4153	0.4524	0.0143	0.0007	0.9532	0.0985	ns	ns	ns
ZT13-14	0.8556	0.8430	0.7427	0.0148	0.8993	0.0020	ns	ns	ns
ZT14-15	0.5386	0.6505	0.7164	0.0193	0.0172	0.0207	ns	ns	ns
ZT15-16	0.6468	0.0463	0.7030	0.0118	0.0470	0.0025	ns	*	ns

ZT16-17	0.3764	0.4060	0.1597	0.0034	0.0234	0.0109	ns	ns	ns
ZT17-18	0.9319	0.6260	0.7423	0.2286	0.9402	0.0132	ns	ns	ns
ZT18-19	0.8229	0.9259	0.9782	0.5236	0.3345	0.4937	ns	ns	ns
ZT19-20	0.9463	0.3941	0.9300	0.4978	0.8826	0.6145	ns	ns	ns
ZT20-21	0.9414	0.5798	0.8807	0.3993	0.6656	0.3446	ns	ns	ns
ZT21-22	0.3539	0.9949	0.9476	0.7408	0.6482	0.4334	ns	ns	ns
ZT22-23	0.2121	0.5568	0.2526	0.1485	0.0365	0.5154	ns	ns	ns
ZT23-24	0.1292	0.0414	0.0952	0.0139	＞0.9999	0.0437	ns	ns	ns
ZT24-25	0.8655	0.7913	0.6770	0.0139	0.0274	0.5071	ns	ns	ns
ZT25-26	0.9087	0.7469	0.7692	0.0170	0.0940	0.0012	ns	ns	ns
ZT26-27	0.1199	0.8552	0.0069	0.0027	0.0055	0.1198	ns	ns	ns
ZT27-28	0.0468	0.1457	0.0236	0.1713	0.0057	0.0069	ns	ns	ns
ZT28-29	0.0013	0.0871	0.0115	0.9998	0.8844	0.3795	ns	ns	ns
ZT29-30	0.0036	0.9752	0.0010	0.6750	0.6980	0.9888	ns	ns	ns
ZT30-31	0.1281	0.8489	＜0.0001	0.9690	0.0034	0.9888	ns	ns	ns

			Group		Time		Interaction	
	Data	DFn, DFd	F	p	F	p	F	p
Figure S2B	Total sleep	2, 87	0.7374	0.4813	196.3	＜0.0001	2.6170	＜0.0001
	Sleep episodes	2, 87	9.0790	0.0003	20.05	＜0.0001	0.8662	0.7583
	P(wake)	2, 87	1.4220	0.2467	181.0	＜0.0001	2.6300	＜0.0001

Dunnett's multiple comparisons

	MB043B/dTrpA1 vs MB043B/+			MB043B/dTrpA1 vs dTrpA1/+			Summary		
	Total sleep	Sleep episodes	P(wake)	Total sleep	Sleep episodes	P(wake)	Total sleep	Sleep episodes	P(wake)
ZT0-1	0.0542	0.0947	0.0895	0.0083	0.0087	0.8686	ns	ns	ns
ZT1-2	0.0773	0.2427	0.5187	0.1517	0.1117	0.1746	ns	ns	ns
ZT2-3	0.3812	0.7501	0.1942	0.1885	0.3468	0.0902	ns	ns	ns
ZT3-4	0.8887	0.9643	0.8913	0.5610	0.4246	0.2496	ns	ns	ns
ZT4-5	0.1860	0.6133	0.3141	0.3599	0.2616	0.9949	ns	ns	ns
ZT5-6	0.0257	0.3878	0.0367	0.0074	0.3989	0.0040	*	ns	*
ZT6-7	0.4763	0.5832	0.3301	0.1268	0.7228	0.4315	ns	ns	ns
ZT7-8	0.4915	0.9954	0.9840	0.1722	0.8730	0.0294	ns	ns	ns
ZT8-9	0.9269	0.0304	0.2414	0.2756	0.7988	0.2011	ns	ns	ns
ZT9-10	0.0431	0.1218	0.1413	0.5599	0.0637	0.3395	ns	ns	ns
ZT10-11	0.0397	0.2360	0.1278	0.0063	0.0454	0.5039	*	ns	ns
ZT11-12	0.3379	0.7433	0.4231	0.0322	0.2603	0.0010	ns	ns	ns
ZT12-13	0.0010	0.0923	0.0232	0.0099	0.6299	0.0261	**	ns	*
ZT13-14	0.3856	＞0.9999	0.1911	0.5492	0.0433	0.3573	ns	ns	ns
ZT14-15	0.8210	0.9296	0.9701	0.9751	0.6404	0.7050	ns	ns	ns
ZT15-16	0.2657	0.4844	0.8947	0.3767	0.6163	0.4584	ns	ns	ns
ZT16-17	0.0167	0.7103	0.1198	0.0211	0.5542	0.2998	*	ns	ns
ZT17-18	0.3970	0.7614	0.1917	0.5029	0.3777	0.2287	ns	ns	ns
ZT18-19	0.4264	0.4994	0.2790	0.8455	0.1745	0.9340	ns	ns	ns
ZT19-20	0.8153	0.8355	0.9636	0.7050	0.0930	0.9907	ns	ns	ns
ZT20-21	0.4155	0.5319	0.8338	0.1225	0.6983	0.2679	ns	ns	ns
ZT21-22	0.7446	0.9676	0.9880	0.6336	0.9920	0.3069	ns	ns	ns
ZT22-23	0.6136	0.4624	0.9295	0.1035	0.9415	0.2829	ns	ns	ns
ZT23-24	0.3903	0.2161	0.5840	0.3318	0.5054	0.1491	ns	ns	ns
ZT24-25	0.3200	0.8207	0.0750	0.2397	0.6446	0.7277	ns	ns	ns
ZT25-26	0.1311	0.9551	0.3670	0.1416	0.8683	0.0840	ns	ns	ns
ZT26-27	0.3947	0.9924	0.1422	0.0485	0.1254	0.1170	ns	ns	ns
ZT27-28	0.7008	＞0.9999	0.4673	0.2948	0.0098	0.0488	ns	ns	ns
ZT28-29	0.1018	0.3762	0.1138	0.9999	0.1909	0.7851	ns	ns	ns
ZT29-30	0.1215	0.0680	0.0808	0.5694	0.1893	0.9435	ns	ns	ns
ZT30-31	0.0503	0.1580	0.0209	0.7486	0.2126	0.8009	ns	ns	ns

			Group		Time		Interaction	
	Data	DFn, DFd	F	p	F	p	F	p
Figure S2C	Total sleep	2, 98	1.0650	0.3487	222.1	＜0.0001	3.7180	＜0.0001
	Sleep episodes	2, 98	2.5120	0.0863	32.28	＜0.0001	1.9580	＜0.0001
	P(wake)	2, 98	0.8850	0.4160	181.4	＜0.0001	5.3520	＜0.0001

			Group		Time		Interaction	
	Data	DFn, DFd	F	p	F	p	F	p
Figure S2D	Total sleep	2, 101	6.0000	0.0034	167.4	＜0.0001	5.2050	＜0.0001
	Sleep episodes	2, 101	13.20	＜0.0001	17.65	＜0.0001	2.5730	＜0.0001

P(wake)			2, 104	2.194	0.1166	135.3	<0.0001	7.3347	<0.0001
	Dunnett's multiple comparisons								
	MB043B/shi^{ts1} vs MB043B/+			MB043B/shi^{ts1} vs shi^{ts1}/+			Summary		
	Total sleep	Sleep episodes	P(wake)	Total sleep	Sleep episodes	P(wake)	Total sleep	Sleep episodes	P(wake)
ZT0-1	0.1800	0.4486	0.0048	0.0103	0.0085	0.1223	ns	ns	ns
ZT1-2	0.9957	0.1574	0.5541	0.1346	0.4120	0.0026	ns	ns	ns
ZT2-3	0.8998	0.0948	0.2824	0.6483	0.5005	0.8577	ns	ns	ns
ZT3-4	0.9987	0.0428	0.9950	0.0070	0.0336	0.2015	ns	*	ns
ZT4-5	0.1434	0.0262	0.5763	<0.0001	0.8067	0.0006	ns	ns	ns
ZT5-6	0.3662	0.9111	0.0273	<0.0001	0.0406	<0.0001	ns	ns	*
ZT6-7	0.0409	0.5251	0.7056	<0.0001	0.9842	<0.0001	*	ns	ns
ZT7-8	0.0334	0.0232	0.0003	0.8532	0.3051	<0.0001	ns	ns	***
ZT8-9	0.0390	0.0486	0.0054	0.9213	0.9037	0.3300	ns	ns	ns
ZT9-10	0.6141	0.7464	0.8216	0.1060	0.8155	0.6512	ns	ns	ns
ZT10-11	0.8941	0.9994	0.5373	0.6583	0.0818	0.0220	ns	ns	ns
ZT11-12	0.2796	0.9921	0.7696	0.0028	0.0009	0.0023	ns	ns	ns
ZT12-13	0.7142	0.8039	0.6344	0.9829	0.7743	0.2227	ns	ns	ns
ZT13-14	0.1356	0.2098	0.5681	0.0129	0.0274	0.2156	ns	ns	ns
ZT14-15	0.0098	0.2677	0.0161	0.0082	0.0048	0.0168	**	ns	*
ZT15-16	0.1530	0.0158	0.0192	0.0623	0.0155	0.0329	ns	*	*
ZT16-17	0.0273	0.0110	0.0684	0.0072	0.0029	0.0475	*	*	ns
ZT17-18	0.0531	0.1402	0.1046	0.0115	0.0153	0.0087	ns	ns	ns
ZT18-19	0.0609	0.0210	0.1614	0.0004	0.0002	0.0038	ns	*	ns
ZT19-20	0.5793	0.0212	0.3301	0.0489	0.0513	0.0029	ns	ns	ns
ZT20-21	0.7019	0.2464	0.9120	0.0485	0.0173	0.3255	ns	ns	ns
ZT21-22	0.9867	0.5995	0.7299	0.0279	0.0600	0.0086	ns	ns	ns
ZT22-23	0.9994	0.8742	0.8509	0.6676	0.1044	0.3311	ns	ns	ns
ZT23-24	0.9922	0.1545	0.4956	0.7800	0.2270	0.9768	ns	ns	ns
ZT24-25	0.0029	0.1390	0.5547	0.0019	0.0072	0.9952	**	ns	ns
ZT25-26	0.0096	0.8586	0.0225	0.0065	0.0013	<0.0001	**	ns	*
ZT26-27	0.0539	0.3434	0.2088	0.5070	0.4646	0.0339	ns	ns	ns
ZT27-28	0.1591	0.3726	0.2383	0.1718	0.2547	0.9173	ns	ns	ns
ZT28-29	0.7048	0.2134	0.1502	0.0149	0.8512	0.1055	ns	ns	ns
ZT29-30	0.8512	0.1013	0.9681	0.0451	0.0135	0.0077	ns	ns	ns
ZT30-31	0.6151	0.6842	0.9183	0.0398	0.2023	0.0615	ns	ns	ns
