# Supplementary material for: Brief disruption of activity in a subset of dopaminergic neurons during consolidation impairs long-term memory by fragmenting sleep
Source: eLife. 2026 Sep 23;14:RP104862. doi: 10.7554/eLife.104862 (PMC13600697; doi:10.7554/eLife.104862)
Supplement: Peer review history

# Editor report: eLife Assessment

**DOI:** 10.7554/eLife.104862.4.sa0

**Authors:** Esteban J Beckwith

**Affiliations:** Instituto de Investigación en Biomedicina de Buenos Aires (IBioBA) - CONICET Argentina

**Keywords:** Important, Solid

This study provides **important** insights into the neural mechanisms linking sleep and long-term memory consolidation. By combining behavioural, genetic, imaging, and connectomic approaches in Drosophila, it identifies a target neural circuit that will be of broad interest to researchers studying sleep, memory, and neural circuits. The evidence supporting the involvement of the identified circuit in the regulation of sleep and memory is **solid** and represents a substantial advance in the field. Nevertheless, there is limited evidence to support the mechanistic claim that this circuit directly links sleep and memory consolidation within the available data, and some results should therefore be interpreted with appropriate caution.

# Referee report: Reviewer #1 (Public review)

**DOI:** 10.7554/eLife.104862.4.sa1

[Editors' note: this version has been assessed by the Reviewing Editor without further input from the original reviewers. The authors have considered and discussed the comments raised in the previous round of review.]

Summary:

The authors aim to use state-of-the art behaviour, imaging and connectome techniques to identify the neural interaction between sleep and long-term memory consolidation in the PAM-DPM circuits, a well-known dopaminergic pathway within Drosophila Mushroom Body.

Strengths:

The investigation follows a logical strategy to collect huge dataset of sleep, appetitive memory and live imaging. The authors identified and showed that activation of a PAM subset: alpha-1 reduces sleep quality and memory consolidation in a starvation dependant manner. The author also convincingly demonstrated the corresponding neuronal responses of DPM neurons following PAM alpha-1 activation, and the positive role of DPM neural activity in sleep and memory consolidation. Moreover, the new data provide TRIC-LUC provided better temporal resolution of neural activity correlates for PAMalpha1-DPM inhibition. Importantly, the author demonstrated that memory loss derived from PAM alpha 1 activation can be partly restored by ectopic sleep enhancement via feeding THIP at the memory consolidation period after training.

Weaknesses:

Although the revised version carries arguments to satisfy the reviewers' concern, the writing is now less cohesive. Crucially an explanation however remains required for the following experimental contradiction: the central observation of the study indicates that PAM alpha1 activation cause DPM inhibition which disrupt sleep and memory consolidation. Therefore, one would expect a reduced PAMalpha1 and increased DPM activities after memory training, but the authors found the opposite is true from now enhanced TRIC-LUC dataset. The authors indicate this data reinforce the inhibitory nature of PAM-alph1-DPM, but it does not explain why such a reduced DPM activity is observed after training.

# Referee report: Reviewer #2 (Public review)

**DOI:** 10.7554/eLife.104862.4.sa2

Summary:

Sleep plays a critical role in memory consolidation, but the neural mechanisms underlying this relationship remain incompletely understood. The authors examined a specific subset of PAM dopaminergic neurons, PAM-α1, and DPM neurons in Drosophila. These neurons have previously been implicated in memory, and DPM neurons have also been linked to sleep. The study explores whether this circuit provides a mechanistic link between sleep and memory consolidation.

Strengths:

The authors report several novel findings. Brief activation or inhibition of PAM-α1 neurons, or brief inhibition of DPM neurons during the first few hours after training, impairs 24-hour LTM. Notably, these brief manipulations disrupt sleep for many hours afterward, particularly during the night. The authors further show that perturbation of PAM-α1 and DPM neurons impairs sleep and appetitive memory consolidation under starvation conditions, and that pharmacological sleep induction during the night rescues the LTM defects. Together, these findings suggest that PAM-α1 and DPM neurons are involved in sleep regulation and LTM consolidation under starvation. These are important observations that advance our understanding of the circuits regulating sleep and memory consolidation.

Weaknesses:

Some claims require additional evidence or clarification.

(1) Previous studies linking impaired memory to reduced sleep have primarily examined conditions involving severe sleep deprivation. In contrast, this manuscript argues that relatively modest decreases in total sleep, accompanied by sleep fragmentation, are sufficient to impair memory consolidation. It remains unclear whether sleep fragmentation of this magnitude is itself critical for LTM consolidation. An independent method for inducing comparably mild sleep loss and fragmentation would be needed to directly test this interpretation.

(2) It is unclear why both activation and inactivation of PAM-α1 neurons produce similar effects on sleep and memory. In addition, MB299B-labeled neurons exert stronger effects on memory than MB043B-labeled neurons, whereas MB043B-labeled neurons have stronger effects on sleep. If sleep disruption is the primary driver of impaired memory consolidation, a stronger correspondence between the sleep and memory phenotypes might be expected. The authors speculate that MB043B may affect sleep through non-PAM neurons, but without identifying the relevant neurons, this remains speculative.

(3) The complex schematic model (Fig. 12), with parallel circuits and unidentified neuronal groups, underscores the difficulty of interpreting the current data. In the "less activity" arm of the model, distinct circuits are proposed to regulate sleep and LTM, respectively, and DPM neurons are not included. This makes it difficult to reconcile the model with the central claim that the PAM-α1-to-DPM microcircuit links sleep and LTM consolidation.

(4) The TRIC-LUC reporter system is not ideal for resolving dynamic changes in neuronal activity. Activity-dependent Ca²⁺ signaling must first reconstitute the TRIC transcriptional system, which then drives luciferase transcription, translation, and accumulation. The original characterization of TRIC indicates that TRIC signals accumulate and decay over several hours. Thus, the kinetics of the TRIC-LUC reporter should be interpreted cautiously, particularly when inferring transient or precisely timed changes in neuronal activity.

(5) Including data from training under fed conditions would provide a more complete understanding of state-dependent neural activity and would help distinguish starvation-specific effects from more general circuit mechanisms.

# Referee report: Reviewer #3 (Public review)

**DOI:** 10.7554/eLife.104862.4.sa3

Summary:

Understanding the neural circuits that link sleep and memory remains a fundamental challenge in neuroscience. In this study, Lin Yan and colleagues investigate how dopamine signaling in Drosophila regulates long-term memory (LTM) formation in the context of sleep. They identify a specific microcircuit between protocerebral anterior medial dopamine neurons (PAM-DANs) and dorsal paired medial (GABAergic DPM) neurons that modulates memory consolidation. Their findings suggest that disrupting the basal activity of PAM-α1 neurons during early consolidation impairs LTM, with particularly pronounced effects under starvation conditions. Notably, sleep fragmentation caused by this disruption can be pharmacologically rescued, restoring LTM. These results provide compelling evidence how dopamine signaling plays a crucial role in linking sleep and memory, offering new insights into the underlying mechanisms.

Strength:

This study presents a well-executed investigation into sleep-memory interactions, utilizing a combination of connectomics, behavioral assays, functional imaging, and pharmacological manipulations. The authors convincingly demonstrate that the PAM-α1 and DPM circuit interact, highlighting a potential mechanism by which sleep influences memory consolidation. The anatomical and functional dissection of this circuit is of high interest to the field, and the study's integration of sleep and memory processes contributes significantly to our understanding of the role of dopamine in cognitive functions. Additional experiments investigating the contribution of MBON-α1 to the circuit, connectomic analysis together with a dissection of dopamine receptor function further strengthen the proposed circuit motif and its biological relevance.

Weaknesses:

While the study is well designed, presents compelling findings and has been further strengthened by additional experiments, some aspects remain unclear. The role of DPM neurons in memory consolidation seems not yet fully resolved, as different genetic approaches yield variable results. Furthermore, some manipulations impair memory without affecting sleep fragmentation - or vice versa, suggesting that the observed memory deficits cannot be explained solely by impaired sleep-dependent consolidation. It would also have been interesting to discuss potential mechanisms by which dopamine receptor-mediated cAMP signaling could lead to a reduction in Ca²⁺ signals. I am confident that these questions can be addressed in future studies.

Conclusion:

Overall, this study provides valuable new insights into how sleep and dopaminergic circuits interact to regulate memory consolidation in Drosophila and may reveal general principles underlying the neural regulation of memory.

# Author comment: Author response

**DOI:** 10.7554/eLife.104862.4.sa4

**Authors:** Lin Yan, Litao Wu, Timothy D Wiggin, Xiaojuan Su, Wei Yan, Hailiang Li, Lei Li, Zhonghua Lu, Fang Guo, Zhiqiang Meng, Yuantao Li, Fan Li, Leslie C Griffith, Chang Liu

**Affiliations:** Shenzhen Institute of Advanced Technology, Chinese Academy of Sciences Shenzhen China; Zhejiang University Hangzhou China; Brandeis University Waltham United States; Shenzhen Institute of Advanced Technology, Chinese Academy of Sciences Shenzhen China; Shenzhen Institute of Advanced Technology, Chinese Academy of Sciences Shenzhen China; Zhejiang University Hangzhou China; Shenzhen Institutes of Advanced Technology Shenzhen China; Shenzhen Institutes of Advanced Technology Shenzhen China; Zhejiang University Hangzhou China; Shenzhen Institute of Advanced Technology, Chinese Academy of Sciences Shenzhen China; Shenzhen Maternity and Child Healthcare Hospital Shenzhen China; Shantou University Medical College Shantou China; Brandeis University Waltham United States; Shenzhen University of Advanced Technology Shenzhen China

The following is the authors’ response to the previous reviews

**eLife Assessment**

This study provides important insights into the neural mechanisms linking sleep and long-term memory consolidation. By combining behavioural, genetic, imaging, and connectomic approaches in Drosophila, it identifies a target neural circuit that will be of broad interest to researchers studying sleep, memory, and neural circuits. The evidence supporting the involvement of the identified circuit in the regulation of sleep and memory is solid and represents a substantial advance in the field. Nevertheless, there is limited evidence to support the mechanistic claim that this circuit directly links sleep and memory consolidation within the available data, and some results should therefore be interpreted with appropriate caution.

We appreciate the reviewer’s careful evaluation of our manuscript, and we agree that (as with any experimental study) there is still a lot to do to fully understand the mechanisms underlying the linkage between sleep and memory consolidation.

**Public Reviews:**

**Reviewer #1 (Public review):**

Summary:

The authors aim to use state-of-the art behaviour, imaging and connectome techniques to identify the neural interaction between sleep and long-term memory consolidation in the PAM-DPM circuits, a well-known dopaminergic pathway within Drosophila Mushroom Body.

Strengths:

The investigation follows a logical strategy to collect huge dataset of sleep, appetitive memory and live imaging. The authors identified and showed that activation of a PAM subset: alpha-1 reduces sleep quality and memory consolidation in a starvation dependent manner. The author also convincingly demonstrated the corresponding neuronal responses of DPM neurons following PAM alpha-1 activation, and the positive role of DPM neural activity in sleep and memory consolidation. Moreover, the new data provide TRIC-LUC provided better temporal resolution of neural activity correlates for PAMalpha1-DPM inhibition. Importantly, the author demonstrated that memory loss derived from PAM alpha 1 activation can be partly restored by ectopic sleep enhancement via feeding THIP at the memory consolidation period after training.

Weaknesses:

Although the revised version carries arguments to satisfy the reviewers' concern, the writing is now less cohesive. Crucially an explanation however remains required for the following experimental contradiction: the central observation of the study indicates that PAM alpha1 activation cause DPM inhibition which disrupt sleep and memory consolidation. Therefore, one would expect a reduced PAMalpha1 and increased DPM activities after memory training, but the authors found the opposite is true from now enhanced TRIC-LUC dataset. The authors indicate this data reinforce the inhibitory nature of PAM-alph1-DPM, but it does not explain why such a reduced DPM activity is observed after training.

We thank the reviewer for their point of view. We have faithfully reported all experimental observations acquired from our enhanced TRIC-LUC dataset as objectively as possible. We note that the reviewer postulates a particular expected activity shift (reduced PAM-α1 activity and elevated DPM activity after memory training), yet it is not clear why. As our data show, this is a highly connected microcircuit and it is not easy to predict how it might change. Our data show that there is change and dismissing our empirically measured results purely based on a theoretical expectation is not justified. The brain operates as an intricately interconnected network; neural activity dynamics cannot always be simply inferred from static circuit polarity. Progress in deciphering neural circuit function relies on iterative rounds of experimental testing. Like most neuroscience investigations, the present study cannot resolve every open question, and we explicitly acknowledge several unresolved directions worthy of future exploration in the Discussion section.

**Reviewer #2 (Public review):**

Summary:

Sleep plays a critical role in memory consolidation, but the neural mechanisms underlying this relationship remain incompletely understood. The authors examined a specific subset of PAM dopaminergic neurons, PAM-α1, and DPM neurons in Drosophila. These neurons have previously been implicated in memory, and DPM neurons have also been linked to sleep. The study explores whether this circuit provides a mechanistic link between sleep and memory consolidation.

Strengths:

The authors report several novel findings. Brief activation or inhibition of PAM-α1 neurons, or brief inhibition of DPM neurons during the first few hours after training, impairs 24-hour LTM. Notably, these brief manipulations disrupt sleep for many hours afterward, particularly during the night. The authors further show that perturbation of PAM-α1 and DPM neurons impairs sleep and appetitive memory consolidation under starvation conditions, and that pharmacological sleep induction during the night rescues the LTM defects. Together, these findings suggest that PAM-α1 and DPM neurons are involved in sleep regulation and LTM consolidation under starvation. These are important observations that advance our understanding of the circuits regulating sleep and memory consolidation.

Weaknesses:

Some claims require additional evidence or clarification.

(1) Previous studies linking impaired memory to reduced sleep have primarily examined conditions involving severe sleep deprivation. In contrast, this manuscript argues that relatively modest decreases in total sleep, accompanied by sleep fragmentation, are sufficient to impair memory consolidation. It remains unclear whether sleep fragmentation of this magnitude is itself critical for LTM consolidation. An independent method for inducing comparably mild sleep loss and fragmentation would be needed to directly test this interpretation.

We appreciate the reviewer’s suggestion. While alternative assays for inducing sleep loss or sleep fragmentation are indeed available, this line of investigation lies beyond the core scope of the present study. We will certainly take this valuable suggestion into consideration for the future studies.

Regarding the question of whether sleep fragmentation of this magnitude per se is critical for long-term memory consolidation, we would like to highlight relevant published evidence. Prior work in rodents and human (Bonnet and Arand, 2003; Van Someren et al., 2015; Ramesh et al., 2012; Baud et al., 2014) and our earlier study (Liu et al., 2019) have demonstrated that alterations in sleep architecture, independent of changes in total sleep amount, can affect multiple physiological processes, including memory. Given this existing supporting evidence, we respectfully argue that this does not constitute a weakness of the present manuscript.

(2) It is unclear why both activation and inactivation of PAM-α1 neurons produce similar effects on sleep and memory. In addition, MB299B-labeled neurons exert stronger effects on memory than MB043B-labeled neurons, whereas MB043B-labeled neurons have stronger effects on sleep. If sleep disruption is the primary driver of impaired memory consolidation, a stronger correspondence between the sleep and memory phenotypes might be expected. The authors speculate that MB043B may affect sleep through non-PAM neurons, but without identifying the relevant neurons, this remains speculative.

The concern raised by the reviewer that certain interpretations remain speculative represents a common situation in most published research. This interesting direction warrants further investigation in future work, but falls outside the scope of the present study. In the revised manuscript, we have elaborated on the differences observed between these two GAL4 drivers. We have also conducted additional experiments to investigate a well-characterized memory-related recurrent loop of PAM-α1 neurons in sleep regulation. We respectfully note that no single study can comprehensively address all outstanding questions.

(3) The complex schematic model (Fig. 12), with parallel circuits and unidentified neuronal groups, underscores the difficulty of interpreting the current data. In the "less activity" arm of the model, distinct circuits are proposed to regulate sleep and LTM, respectively, and DPM neurons are not included. This makes it difficult to reconcile the model with the central claim that the PAM-α1-to-DPM microcircuit links sleep and LTM consolidation.

We appreciate this careful comment on our schematic model in Figure 12. This diagram aims to summarize the key findings obtained in the present study while also explicitly laying out unresolved questions that await future investigation. In our view, including open, outstanding questions in the working model does not undermine the interpretation of our existing experimental results. In the revised manuscript, we have modified the corresponding text to clarify this point and distinguish firmly between conclusions supported by our data and tentative components requiring follow-up validation.

(4) The TRIC-LUC reporter system is not ideal for resolving dynamic changes in neuronal activity. Activity-dependent Ca^2+^ signaling must first reconstitute the TRIC transcriptional system, which then drives luciferase transcription, translation, and accumulation. The original characterization of TRIC indicates that TRIC signals accumulate and decay over several hours. Thus, the kinetics of the TRIC-LUC reporter should be interpreted cautiously, particularly when inferring transient or precisely timed changes in neuronal activity.

We fully acknowledge the inherent limitations of the TRIC-LUC reporter system, as pointed out by the reviewer. Every experimental tool comes with characteristic strengths and drawbacks. Although TRIC-LUC suffers from temporal delays, our experiment does not aim to capture acute, immediate effects; instead, it examines long-term dynamics of neuronal activity. To date, within Drosophila neurobiology, no superior technique is available for non-invasive long-term monitoring of neuronal activity in freely behaving flies. We share the hope that new tools capable of reporting neuronal activity in real time will be developed and applied, which will facilitate deeper mechanistic understanding of neuronal dynamics.

(5) Including data from training under fed conditions would provide a more complete understanding of state-dependent neural activity and would help distinguish starvation-specific effects from more general circuit mechanisms.

We appreciate this suggestion. First, our memory paradigm relies on reward-based associative learning, and starvation is required for flies to express robust memory, so to do this would require a completely new experimental set up. Second, our core findings demonstrate that transient perturbations of this neuronal circuit trigger sleep disturbances and memory deficits specifically under starvation conditions. Therefore, measurements of neural activity under fed conditions are not directly relevant to the central conclusions of the present study. We agree that related experiments on other behaviors under fed states constitute an interesting direction and could be pursued in future investigations.

**Reviewer #3 (Public review):**

Summary:

Understanding the neural circuits that link sleep and memory remains a fundamental challenge in neuroscience. In this study, Lin Yan and colleagues investigate how dopamine signaling in Drosophila regulates long-term memory (LTM) formation in the context of sleep. They identify a specific microcircuit between protocerebral anterior medial dopamine neurons (PAM-DANs) and dorsal paired medial (GABAergic DPM) neurons that modulates memory consolidation. Their findings suggest that disrupting the basal activity of PAM-α1 neurons during early consolidation impairs LTM, with particularly pronounced effects under starvation conditions. Notably, sleep fragmentation caused by this disruption can be pharmacologically rescued, restoring LTM. These results provide compelling evidence how dopamine signaling plays a crucial role in linking sleep and memory, offering new insights into the underlying mechanisms.

Strength:

This study presents a well-executed investigation into sleep-memory interactions, utilizing a combination of connectomics, behavioral assays, functional imaging, and pharmacological manipulations. The authors convincingly demonstrate that the PAM-α1 and DPM circuit interact, highlighting a potential mechanism by which sleep influences memory consolidation. The anatomical and functional dissection of this circuit is of high interest to the field, and the study's integration of sleep and memory processes contributes significantly to our understanding of the role of dopamine in cognitive functions. Additional experiments investigating the contribution of MBON-α1 to the circuit, connectomic analysis together with a dissection of dopamine receptor function further strengthen the proposed circuit motif and its biological relevance.

Weaknesses:

While the study is well designed, presents compelling findings and has been further strengthened by additional experiments, some aspects remain unclear. The role of DPM neurons in memory consolidation seems not yet fully resolved, as different genetic approaches yield variable results. Furthermore, some manipulations impair memory without affecting sleep fragmentation - or vice versa, suggesting that the observed memory deficits cannot be explained solely by impaired sleep-dependent consolidation. It would also have been interesting to discuss potential mechanisms by which dopamine receptor-mediated cAMP signaling could lead to a reduction in Ca^2+^ signals. I am confident that these questions can be addressed in future studies.

We greatly appreciate the reviewer’s positive evaluation of our work and the thoughtful suggestions regarding future directions. As acknowledged in the manuscript, our study centers on identifying a shared circuit that coregulates sleep and memory processes. We have performed preliminary investigations of downstream circuitry, and our results indeed support the idea that sleep and memory can be modulated independently. Importantly, we have avoided drawing definitive conclusions that memory deficits arise purely from impaired sleep-dependent consolidation. Instead, we emphasize the existence of a common circuit mechanism governing both processes.

We also thank the reviewer for drawing attention to dopamine receptor-mediated cAMP signaling and Ca^2+^dynamics. This observation constitutes an additional finding that requires more extensive mechanistic follow-up. Given the scope of the current work, we have not dedicated a separate discussion section to dissecting this pathway. This promising line of inquiry will be pursued in our future research.

Conclusion:

Overall, this study provides valuable new insights into how sleep and dopaminergic circuits interact to regulate memory consolidation in Drosophila and may reveal general principles underlying the neural regulation of memory.

**Recommendations for the authors:**

**Reviewer #1 (Recommendations for the authors):**

No further issue, apart from the reverse figure 13 are not found in the main text as indicated.

We thank the reviewer for this careful check. We have performed a full-text search for Figure 13 throughout the revised manuscript and found no relevant citation. We have also carefully cross-checked all figure numbering and confirm that all figure labels are accurate in the current version.

**Reviewer #2 (Recommendations for the authors):**

In Fig. 8B, some individual GCaMP measurements show values below −100% ΔF/F₀. Under the stated definition, ΔF/F = (Fn - F0) / F0, values below −100% would require Fn to be negative. Since raw fluorescence intensity cannot be negative, values below −100% require further explanation. The authors should clarify whether Fn represents raw fluorescence or processed fluorescence, and whether the plotted traces underwent any normalization, subtraction, detrending, or transformation beyond the stated formula.

We greatly appreciate the reviewer’s rigorous scrutiny of our data and the valuable question raised. Our fluorescence signals were calculated using the standard formula ΔF/F = (Fn − F0)/F0, where Fn = F_ROI − F_background, and all calculations were implemented accordingly. We have carefully revisited all raw imaging datasets and identified the source of the issue. During initial data processing, we retained all acquired recordings without excluding samples exhibiting focal plane drift. This drift occasionally yielded negative values for Fn (F_ROI − F_background). Beyond the five DPM cell bodies from four brains in Figure 8B highlighted by the reviewer, we further detected six additional DPM cell bodies from four brains in Figure 2A affected by the same artifact. We have now excluded these drifting preparations, regenerated all corresponding plots, and updated the statistical analyses in the revised manuscript. For transparency, we upload both the raw and processed datasets as supplementary materials to clarify this point.
